# Pilot Scale Electrolysis of Peroxodicarbonate as an Oxidizer for Lignin Valorization

**DOI:** 10.1021/acssuschemeng.4c02898

**Published:** 2024-07-18

**Authors:** Theresa Rücker, Torbjørn Pettersen, Hannah Graute, Bernd Wittgens, Tobias Graßl, Siegfried R. Waldvogel

**Affiliations:** ‡ Process Technology, SINTEF Industry, Trondheim, Trøndelag NO-7465, Norway; ¶ Karlsruhe Institute of Technology, Kaiserstraße 12, 76131 Karlsruhe, Germany; § CONDIAS GmbH, Fraunhofer Straße 1b, 25524 Itzehoe, Germany; † Max Planck Institute for Chemical Energy Conversion, Stiftstraße 34-36, 45470 Mülheim an der Ruhr, Germany

**Keywords:** flow electrolyzer, peroxodicarbonate, oxidation, lignin, scale-up, process design

## Abstract

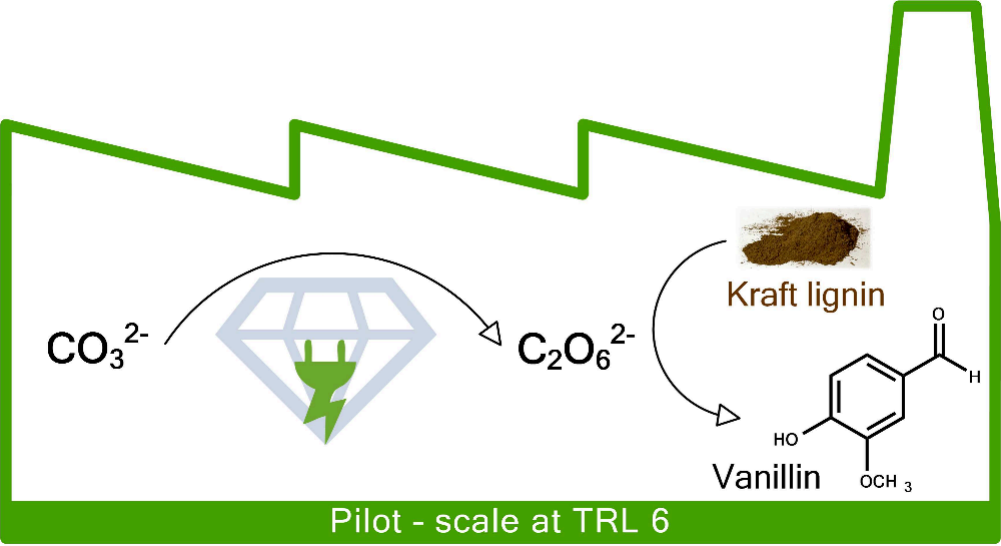

A pilot scale plant at Technology Readiness Level (TRL) 6 comprising an electrochemical ex-cell continuous production of sodium peroxodicarbonate and a thermal depolymerization plug flow reactor for kraft lignin conversion is established. Due to the labile nature of the “green” oxidizer peroxodicarbonate, special attention must be paid to the production parameters in order to optimize its use. A simplified design model describing steady-state and transient operations is formulated and finally validated against experimental data from the electrolysis setup. Design trade-offs are visualized, and their impact on specific energy consumption is evaluated. The pilot plant was operated for a 20-month period for more than 1200 h on-stream. Optimized process conditions result in vanillin yields of 8 wt % and thus prove the successful scale-up.

## Abstract 2

A pilot plant for the continuous, electrochemical ex-cell production of sodium peroxodicarbonate for subsequent use in the thermal depolymerization of Kraft lignin was developed, achieving vanillin yields of 8 wt %.

## Introduction

**Figure 1:**
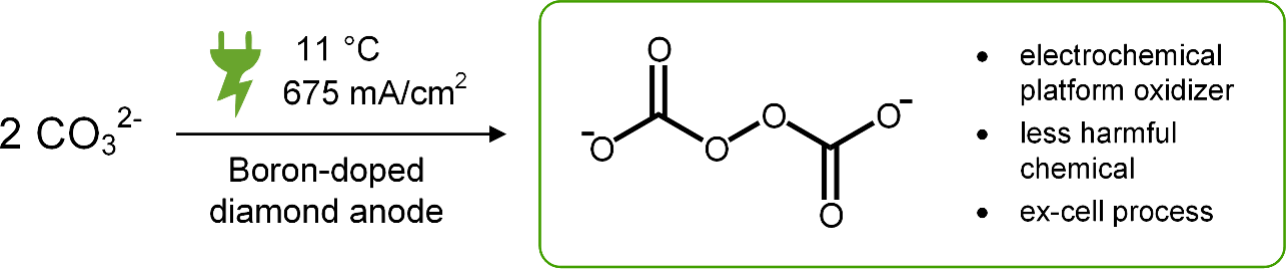
Continuous electrochemical synthesis of peroxodicarbonate at a boron-doped diamond anode with an undivided cell.

Currently, petroleum serves as the predominant feedstock for the industrial production of organic chemicals.^1^ This has tipped over into excessive exploitation of resources and clearly impacts negatively on climate change and the associated increase in greenhouse gas emissions worldwide.^2^ Various economic, political, and social factors are now prompting the chemical industry to explore the utilization of plant-based resources alongside or as alternatives.^3^ Lignocellulosic biomass, as the most abundant biogenic resource, has a huge potential to contribute to the sustainable production of chemicals and fuels.^4^ Historically, the utilization of this biomass has mainly focused on the use of cellulose for paper production with the remaining lignin being incinerated for heat production and recovery of inorganic process chemicals. In particular during the Kraft pulping process, the structure of the lignin changes immensely, making it difficult to process it further into value-added products.^5^ For this reason, a new approach called “lignin-first” or “reductive catalytic fractionation” has been adopted.^6^ These methods depolymerize the lignin from native biomass and stabilize it at the same time to avoid condensation reactions.^7^ Lignin and its derivatives can be used in many valuable applications like adhesives, dispersants, surfactants, bio plastics, composites, bio asphalt, and fuels, as well as low molecular weight compounds in fine and aroma chemicals and fragrances.^8−11^ Converting lignin into value-added products has been investigated using numerous technologies, such as depolymerization reactions.^5,12−17^ One of these is electro-organic synthesis, which has been attracting increasing attention over the past decade since only electrons are used as reagent and thus toxic and/or harmful reagents can be omitted.^18−26^ Additionally, this can save costs compared to convential synthesis.^27^ Both pathways, direct electrolysis^5,28−30^ and via an ex-cell oxidizer,^31−33^ have been studied. The latter can be sodium peroxodicarbonate. It consists of two carbonate moieties which are coupled via a peroxide moiety (Figure 1) and was first electrochemically generated by Riesenfeld et al.^34^ Peroxodicarbonate has low thermal stability and decomposes at room temperature with a half-life of 69 min.^35^ The resulting waste of sodium peroxodicarbonate can be used as a makeup chemical in the paper industry with the nonconverted lignin residues.

After less attention was paid to peroxodicarbonate for almost a century, several research groups focused on its synthesis again at the beginning of the 21st century. Both platinum and boron-doped diamond electrodes were used, and maximum concentrations of 0.5 M could be achieved.^31,36−45^ First, in 2022, Waldvogel and co-workers demonstrated the production of concentrated (0.9 M) peroxodicarbonate solutions using an advanced and sophisticated cell design, based on a copper casing as a heat sink including a microchannel structure.^45^ Besides high current densities above 3 A/cm^2^, a mixture of different cations was used as the electrolyte. However, this approach hampers the possible further use of the carbonate solution as a makeup chemical. As a platform oxidizer, peroxodicarbonate has tremendous potential for technical applications since it can be cogenerated as an alternative anodic reaction to oxygen evolution within the electrochemical water splitting. This is, in particular, of interest when intermittent electricity can be employed. These peroxodicarbonate solutions are safe to use since keeping them at elevated temperature decomposes the peroxide species into oxygen, providing a peroxide-free fraction for downstream processing. Alkali carbonates serve as starting materials, are relatively easy to store, and are safe to handle. Peroxodicarbonate seems to be the ideal reactant for transformations with peroxide wherein alkali base is required. Consequently, this system seems to be made for Kraft lignin degradation.^31^ Recently, peroxodicarbonate has displayed its superior performance in the oxidative conversion of boronic acids,^46^ Dakin reaction,^47^ formation of N-oxides,^48,49^ epoxidation reaction, and sulfur oxidation.^45^ Additionally, it was employed for the bleaching of wood.^44^

In this work, a pilot scale plant consisting of ex-cell continuous production of peroxodicarbonate and a thermal depolymerization plug flow reactor for lignin conversion is presented. A simplified design model is formulated to investigate design options for the reactor setup for continuous peroxodicarbonate production, also describing steady-state and transient operations. Furthermore, the performance of the lignin depolymerization is examined. Various process parameters and the effects of scale-up on the yield of monomeric compounds are discussed.

## Results and Discussion

### Reactivity of Peroxodicarbonate

To develop an understanding of the reactivity of peroxodicarbonate and similar molecules, density functional theory calculations serve as an initial guide, allowing us to derive and quantify relevant parameters. The geometry optimization of peroxodicarbonate, peroxohydrogendicarbonate, and peroxide was performed with the B3LYP hybrid functional and the def2-TZVPP (def2-JK) basis set. The geometrical analysis and graphical representation of the peroxodicarbonate anion reveal the expected nonplanarity in the C–O–O–C substructure, with a corresponding dihedral angle of 90.3° (Table 1). Additionally, the Mayer bond order of the peroxo group is 0.80 (compared to O_2_^2–^: 0.83).

**Table 1:** Molecular Properties

	C_2_O_6_^2**–**^	HC_2_O_6_^–^	O_2_^2–^
*d*(OO)/Å	1.447	1.452	1.541
∠(COOC)/°	90.3	89.3	n.a.
∠(COO)/°	113.4	114.6^a^, 112.5^b^	n.a.
Mayer bond order (OO)	0.80	0.80	0.83

a Protonated group.

b Unprotonated group.

Peroxohydrogendicarbonate shows an unusually high dipole moment. This can be an indication that such a monoanion might not be stable in solution, even if other carbonate species such as ethylene carbonate exhibit high dipole moments of around 4.9 D and are stable. It is important to emphasize that the protonated form of peroxodicarbonate, in particular, was not experimentally observed.

**Figure 2:**
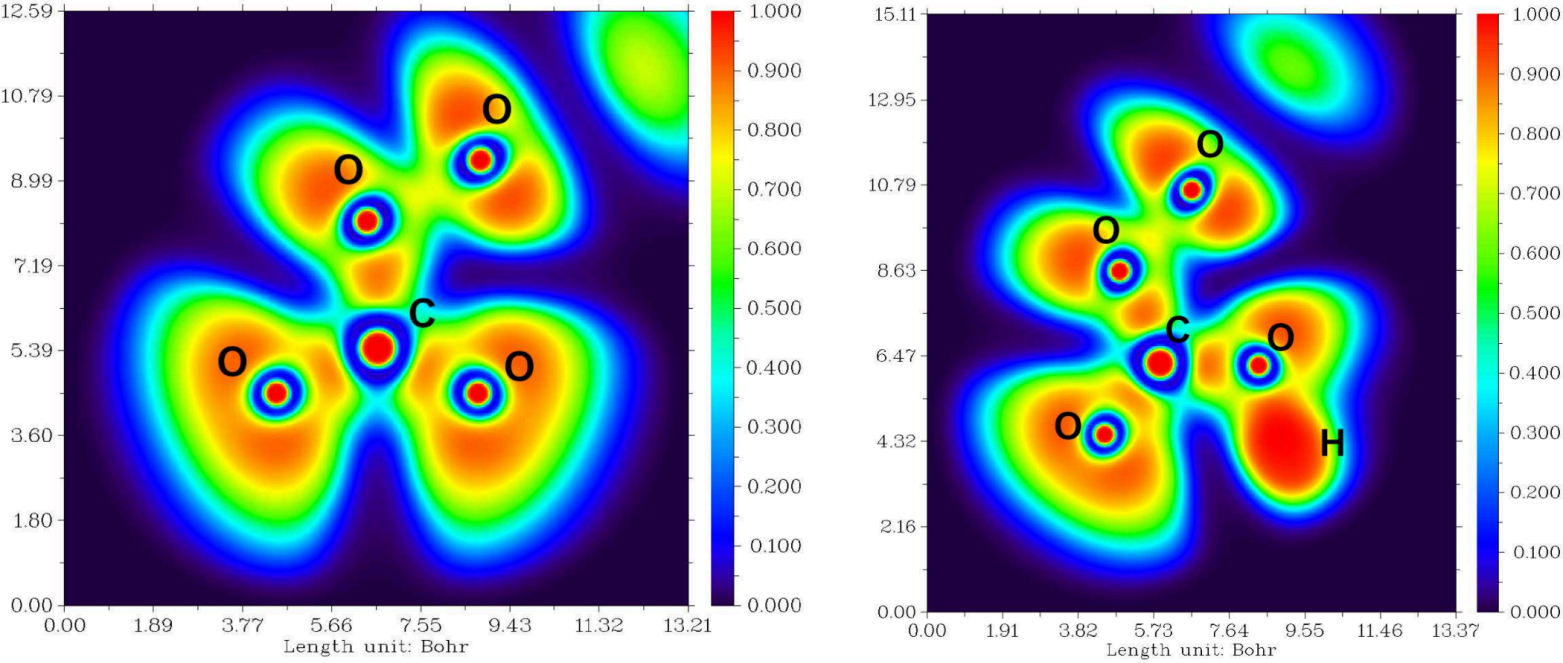
2D heat map representation of the ELF values of peroxodicarbonate (left) and peroxohydrogendicarbonate (right).The cutting plane for both figures was set by the peroxo group and on the neighboring carbon atom, which means that the second carbon atom is not in plane.

The electron localization function (ELF) of peroxodicarbonate and peroxohydrogendicarbonate is displayed in Figure 2 as a 2D heat map.

**Figure 3:**
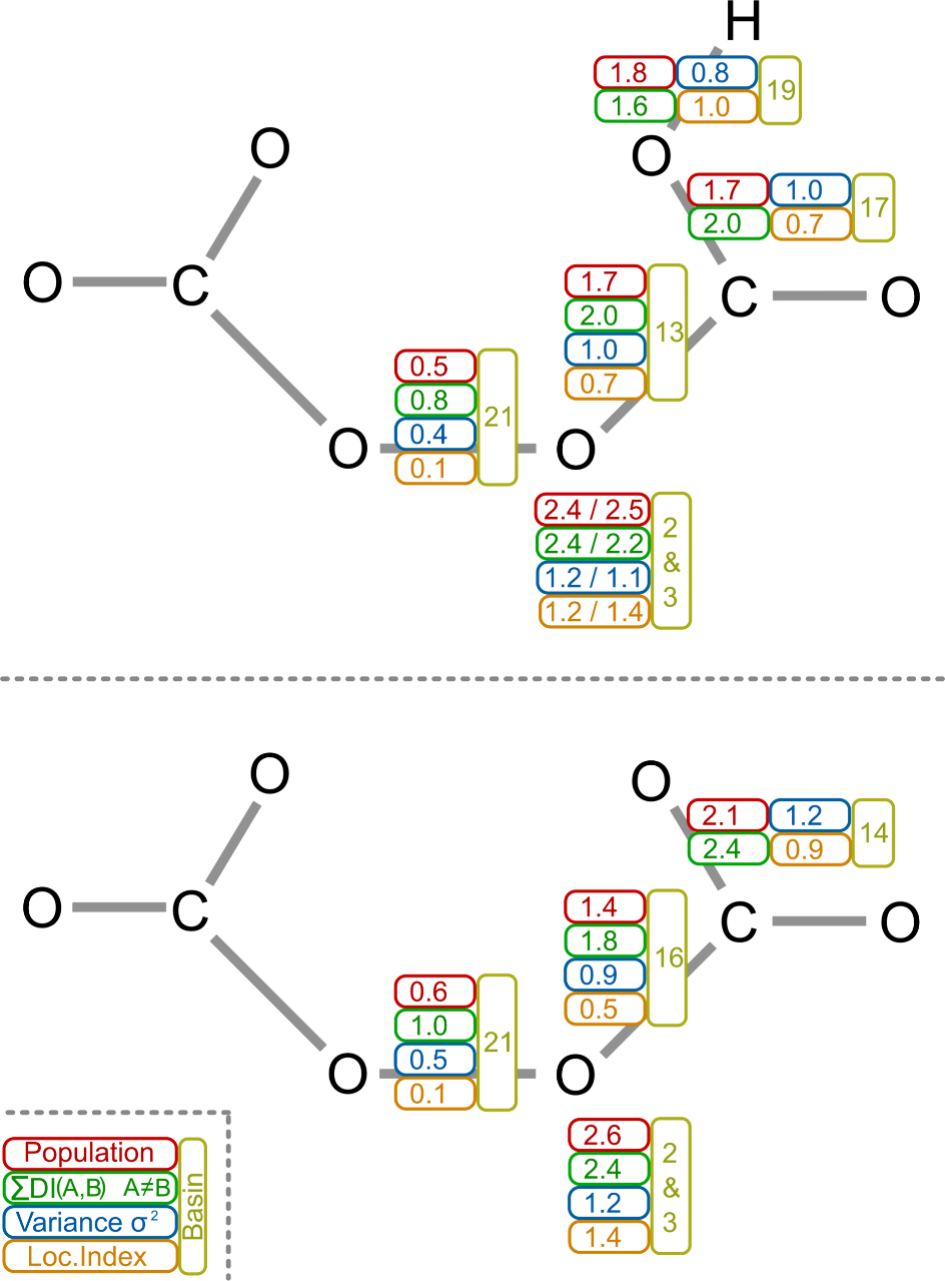
Electron population, localization, and delocalization indices and variances of relevant basins of peroxodicarbonate (left) and peroxohydrogendicarbonate (right). The value boxes are placed on the corresponding bonds (13, 14, 16, 17, 19, and 21) and/or the corresponding lone pair (2 and 3).

The cutting plane was set by the peroxo group and one neighboring carbon atom. Hence, the second carbon atom is not in the plane. The red color indicates a high ELF value, and especially the core basins of oxygen and carbon can be clearly seen. The peroxo bond has a significantly reduced ELF value in both components (Figure 3).

This is expected, as the carbonate substructures draw a large amount of electron density for an sp^2^ system. The electron populations in relevant mono- and disynaptic basins are summarized in Table 2.

**Table 2:** ELF Properties^a^

	C_2_O_6_^2–^	HC_2_O_6_^–^	O_2_^2–^
Population basin O#O	0.6	0.5	0.37
Population basin O–O#C	1.4, 1.4	1.2^b^, 1.7^c^	n.a.
Population basin O–O (LP)	2.6, 2.6	2.4^b^, 2.5^b^	n.a.
Population basin C#O (term.)	2.1	1.7^b^	n.a.
Population basin C#H	n.a.	1.8	n.a.

a Relevant basins marked with “#”.

b Protonated group.

c Unprotonated group. LP: lone pair. Term.: terminal.

The electron population of the disynaptic O–O basins is in accordance with the low calculated Mayer bond order below 1. The Mayer bond order of peroxide is only slightly higher compared to that of the percarbonate species and might indicate a shorter bond length. Instead, the O–O bond of peroxide is longer than in the percabonate species, which in turn is in full agreement with the low electron density in the disynaptic valence basin (O–O). This finding is counterintuitive as it suggests a higher reactivity of peroxide in strongly alkaline media, but the opposite is found experimentally. Hence, the full reactivity pathway has to be investigated experimentally as the calculation indicates that CO_2_ evolution might be a major driving force.

As shown in Table 2, the localization index of the O–O basin is relatively low, i.e., 1/6 and 1/5, respectively, of the population and indicates a very strong electron fluctuation. Nevertheless, the population in the peroxide anion is 0.37, and the localization index is 0.05 which is much lower compared to the carbonate species and supports the statement that the overall reactivity of the peroxodicarbonate species might be mainly driven by the decomposition products.

### Electrochemical Reactor

**Figure 4:**
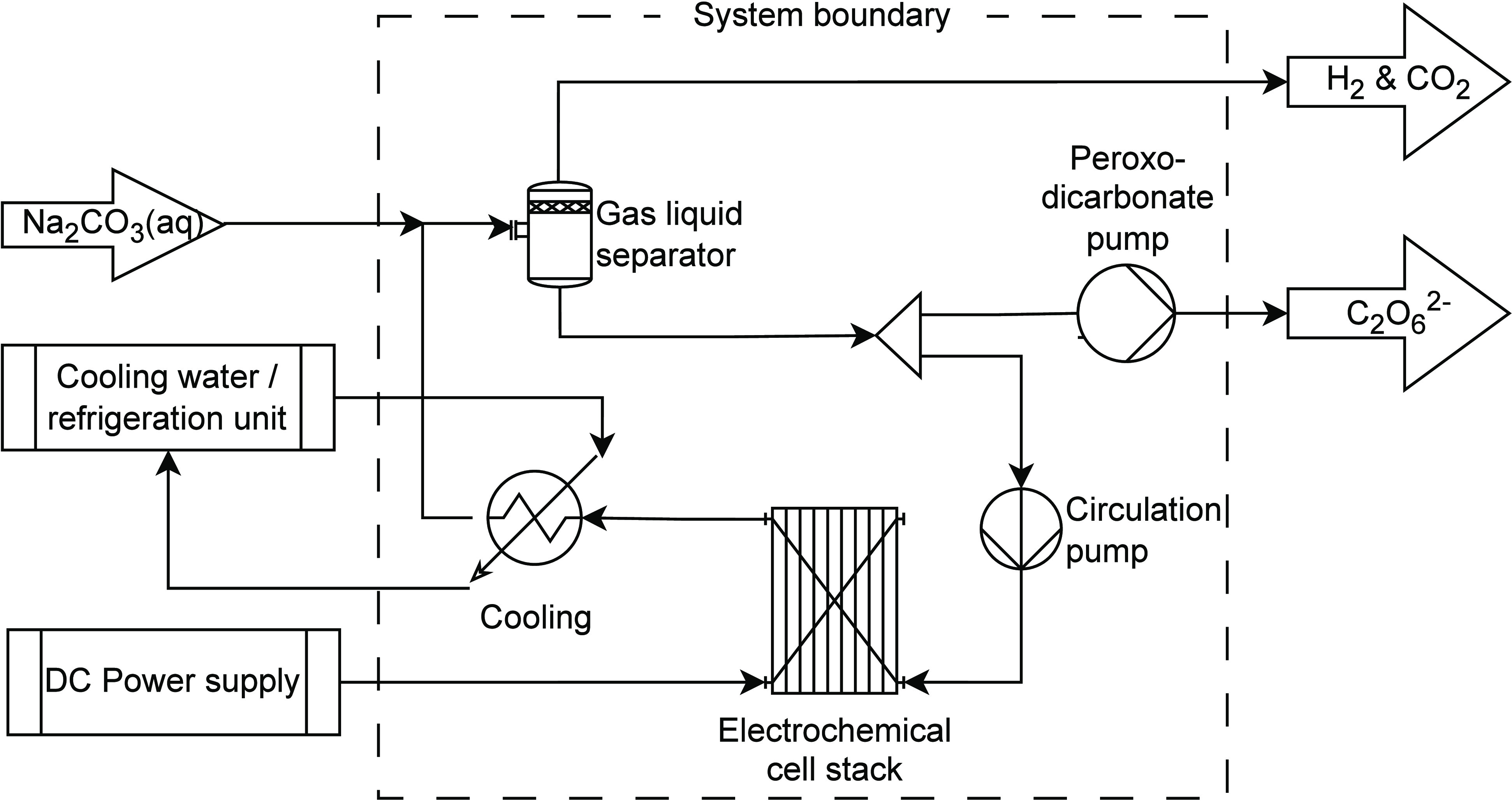
Continuous reactor setup for production of peroxodicarbonate. The system boundary for the simulation model is indicated.

The experimental setup for the continuous production of peroxodicarbonate (Figure 4) consists of a flow loop where the electrolyte is circulated with a constant flow rate through an undivided electrochemical cell, followed by a heat exchanger for cooling, and finally a gas–liquid separator wherein evolved gases are vented. The sodium carbonate feed stream enters between the heat exchanger and the inlet to the gas–liquid separator. The gas–liquid separator provides an extra functionality allowing the reaction mixture to be passed multiple times through the electrochemical cell to build up the peroxodicarbonate concentration to a desired level while maintaining optimal flow conditions within the flow electrolyzer unit. In this pilot setup, fresh makeup Na_2_CO_3_ was allowed to flow into the circulation loop by simple gravitation from a feed storage tank.

The undivided electrochemical cell was provided by CONDIAS GmbH and consists of a single boron-doped diamond anode with characteristic properties given in Table S3. Cooling downstream of the electrochemical cell was provided by a fusion-bonded plate heat exchanger with a cooling capacity of 3 kW. The temperature of the cooling water was kept above 8 °C to avoid the precipitation of sodium carbonate.

### Conceptual Design of the Plant

**Figure 5:**
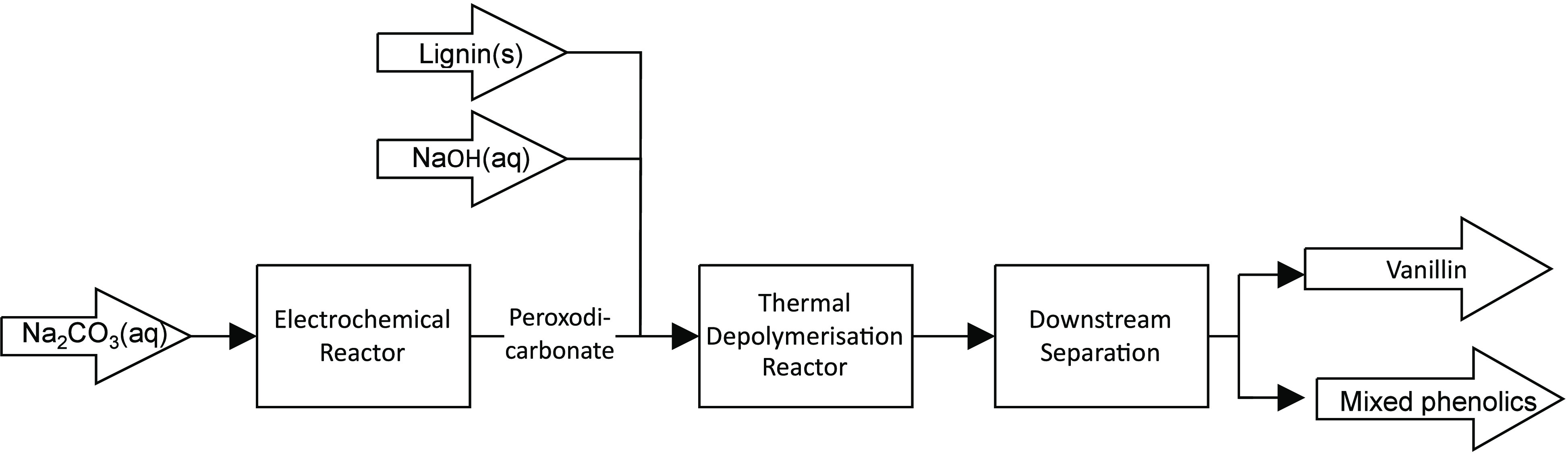
Conceptual design of the lignin depolymerization plant consisting of an electrochemical reactor, a thermal depolymerization reactor, and a downstream separation section.

The pilot plant was designed and built at TRL 6 (technology demonstrated in relevant environment/industrially relevant environment in the case of key enabling technologies). The plant consists mainly of three parts: an electrochemical reactor, a thermal depolymerization reactor, and a downstream separation section (Figure 5). In the first mentioned, an ex-cell approach is chosen for implementation, in which the oxidizing agent peroxodicarbonate is produced via electrolysis in a separate process and made available to the main reaction of lignin depolymerization.

The thermal depolymerization reactor is a plug flow reactor consisting of five segments (inner diameter 102 mm, length 2000 mm), each of which is inclined upward by 3° in the direction of flow to inhibit the possible accumulation of gas that can occur during the reaction. Integrated static mixers ensure axial mixing in a laminar flow and improve the uniform heating of the liquid. The total capacity of the reactor is 80 L. The reactor is equipped with 2 heating zones (1900 W each via segments 1 + 2 and via segments 3 + 4). Temperature sensors in the middle of each reactor segment enable a temperature profile to be monitored along the entire length of the reactor. In addition, there are product sampling openings in each segment, making it possible to take samples at five points simultaneously.^50^

The product separation and purification parts consist of several modularized semicontinuous processes. An absorber system from MionTec GmbH is used for the selective recovery of vanillin and other phenolic monomers from the product stream of the reactor. Several evaporation units, e.g., from ILUDEST Destillationsanlagen GmbH, are used to recover solvents. The pilot unit was operated over a time period of 20 months for more than 1200 h on-stream. The longest periods in continuous operation were typically 100 h, from Monday morning until Friday afternoon.

### Simulation Results

A semiempirical design model was formulated describing the continuous reactor setup (Figure 4) and will be used to investigate key design trade-offs, including transient behavior. The model formulation is based on the work of Chardon et al.^35,42^ on the electrosynthesis of sodium peroxodicarbonate (Na_2_C_2_O_6_) using boron-doped diamond (BDD) anodes and stainless steel cathodes. The reaction mechanism is based on the anodic oxidation of the carbonate salt. This reaction is favored by the properties of BDD electrodes and suppresses the formation of oxygen, as is the normal anodic reaction in alkaline water electrolysis. Hydrogen is formed at the stainless steel cathode.^44^ Therefore, with sodium carbonate (Na_2_CO_3_) as feedstock, the overall reaction for electrochemical production of Na_2_C_2_O_6_ is denoted in eq 1.
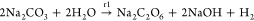
1The production rate (r1) for peroxodicarbonate in the electrochemical cell is proportional to the electrical current (*I*) applied and the Faraday efficiency (η) of the cell (eq 2). The latter was calculated following investigations from Charon et al., who measured the Faraday efficiency as a function of current density.^42^ Here, *z* represents the number of electrical charge, and *F* represents the Faraday constant.
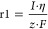
2Once formed, peroxodicarbonate undergoes various decomposition pathways. Zhang and Oloman argue that hydrolysis of sodium peroxodicarbonate is the dominant decomposition reaction that produces hydrogen peroxide and sodium bicarbonate.^39^ Hydrogen peroxide may further decompose into water and oxygen. In the current work, no effort was made to measure the concentrations of H_2_O_2_ and Na_2_C_2_O_6_ individually, as the iodometric titration analysis determines the sum of both components in the reaction mixture. Therefore, based on the level of detail available in the experimental data, a reasonable simplification of the model is to consider the overall reaction forming bicarbonate. This will affect the carbonate equilibrium in the electrolyte and may lead to the release of carbon dioxide if the electrolyte is saturated with CO_2_. Thus, based on the assumption of CO_2_ saturation in the electrolyte, the overall reaction for peroxodicarbonate decomposition is formulated as in eq 3:
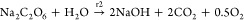
3The reaction rate r2 in eq 3 is defined as a function of the temperature (*T*), the total liquid holdup (*V*), the frequency factor for peroxodicarbonate decomposition (*A*_2_), the activation energy for peroxodicarbonate composition (*E*_2_), the ideal gas constant (*R*), and the concentration of Na_2_C_2_O_6_ (*c*_Na_2_C_2_O_6__) (eq 4).
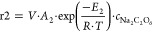
4

#### Simplified Assumptions

Based on the above model formulation, a steady state and dynamic model describing the reactor system (Figure 4) can be formulated. A detailed description is given in the Supporting Information. The following simplifying assumptions are also introduced in the model implementation:

1. Perfect mixing in the gas–liquid separator: The internal recirculation rate through the reservoir and the electrochemical cell is much larger than the overall liquid flow rate through the flow loop.
2. Only hydrogen is present on the cathode: The degradation eq 3 takes place in the gas–liquid separator, which makes up the major part of the liquid hold up of the system.
3. Isothermal operation: The internal recirculation stream between the reservoir and the electrochemical cell is equipped with a heat exchanger with sufficient cooling capacity. Negligible heat loss is assumed from the electrochemical cell and the reservoir. High internal recirculation rates should ensure close-to-isothermal operation.
4. Negligible water evaporation: The amount of water removed via gas venting is assumed negligible compared to the flow rate of water in the Na_2_CO_3_ feed stream.

#### Model Validation

##### Steady-State Model

The model parameters were fitted to experimental data generated by means of the electrochemical reactor. These were collected under constant operating conditions, which were set to a temperature after the cooler of 11 °C, a sodium carbonate feed concentration of 1 M, and a circulation flow rate of 14.5 L/min. Data from steady-state operating conditions (defined as a sample taken after more than 3 h from the last change in set point) were used to fit the electrochemical cell efficiency factor, the peroxodicarbonate degradation frequency factor, and the effective conductivity of the electrolyte solution. The model parameters were fitted by solving the following least-squares optimization problem for the relative deviation between the model prediction and experimental data for the peroxodicarbonate concentration and the cell voltage. The relative error between the predicted and measured peroxodicarbonate concentration has a mean value below 10%, while the mean error in the predicted cell voltage is less than 1%. Solving the least-squares minimization results in a number of model parameters, which are all found to significantly describe the variance in the model data. The parameter correlation matrix shows a moderate correlation between the model parameters related to the Na_2_C_2_O_6_ production and degradation. The strongest correlation is between the two empirical parameters describing the relationship between the cell voltage and cell current. Noteworthily, these parameter sets are not correlated, which is obvious from the model formulation. There is no impact of cell voltage on the Na_2_C_2_O_6_ production rate or the decomposition rate. In reality, the cell voltage might have an impact on the production rate though.

##### Dynamic Model

**Figure 6:**
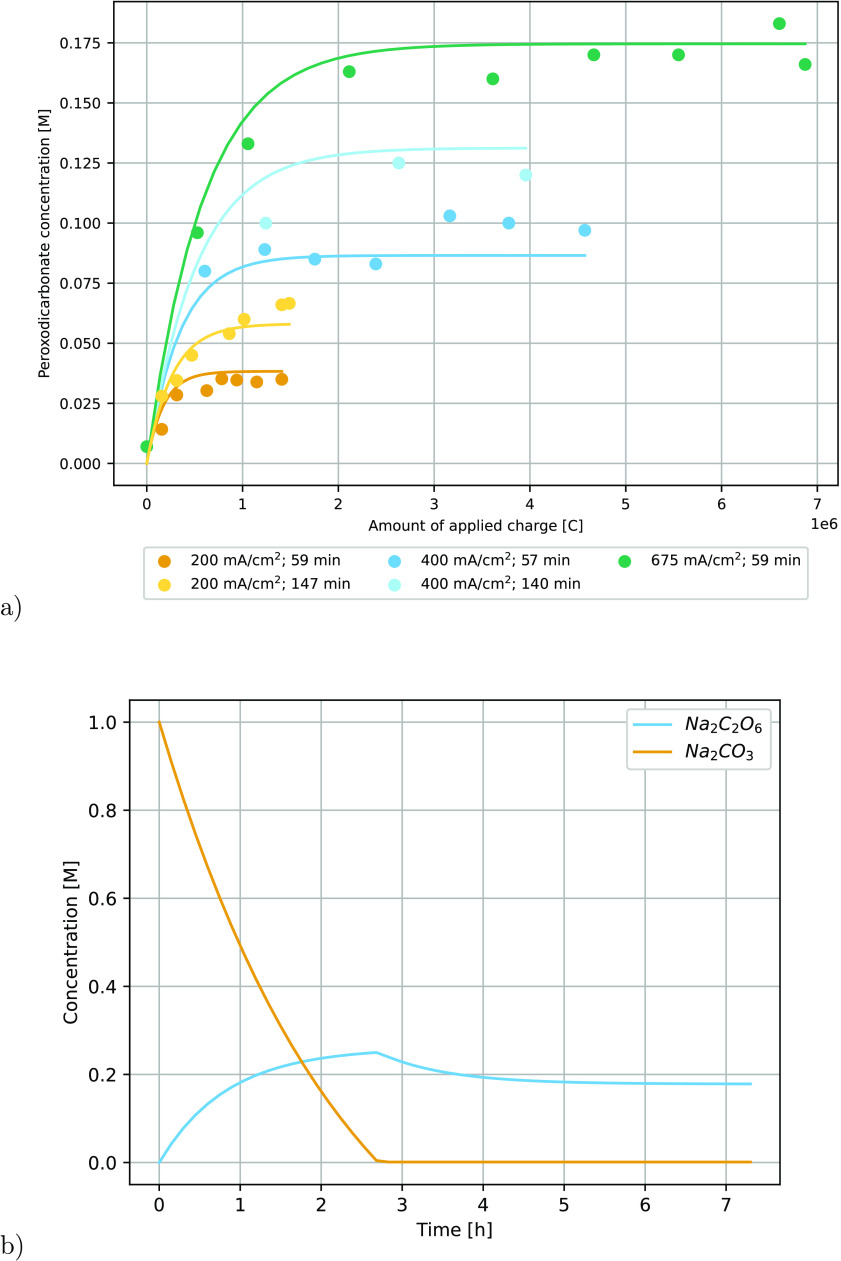
Transient simulation for (a) peroxodicarbonate concentration as a function of time since start-up for varying current densities and time. (b) Consumption of Na_2_CO_3_ and generation of peroxodicarbonate in the reservoir reaching full conversion using 675 mA/cm^2^ and 140 min as the liquid residence time.

To investigate the transient behavior of the peroxodicarbonate concentration in a single reservoir reactor system, a simplistic dynamic model was formulated, taking both feed and product stream into account. This can be used to calculate the evolution of peroxodicarbonate as a function of time since the start of electrochemical production related to the current density and the time of liquid residence in the reservoir during each transient period (Figure 6a). The solid lines with colors matching those of the experimental series are model predictions based on the dynamic model. The results show good agreement. The predictions of the model indicate a maximum in peroxodicarbonate concentration between 2 and 3 h upon the start of electrolysis. The amount of charge applied for 2.5 h was 0.3–2.5 *F* depending on the flow rate and current density used. The case when operating at maximum current density (675 mA/cm^2^) and long liquid residence time in the reservoir (140 min) is not displayed, as a complete conversion of sodium carbonate is achieved. Once the complete conversion of sodium carbonate is accomplished, the concentration of peroxodicarbonate drops (Figure 6b).

#### Design Trade-offs

**Figure 7:**
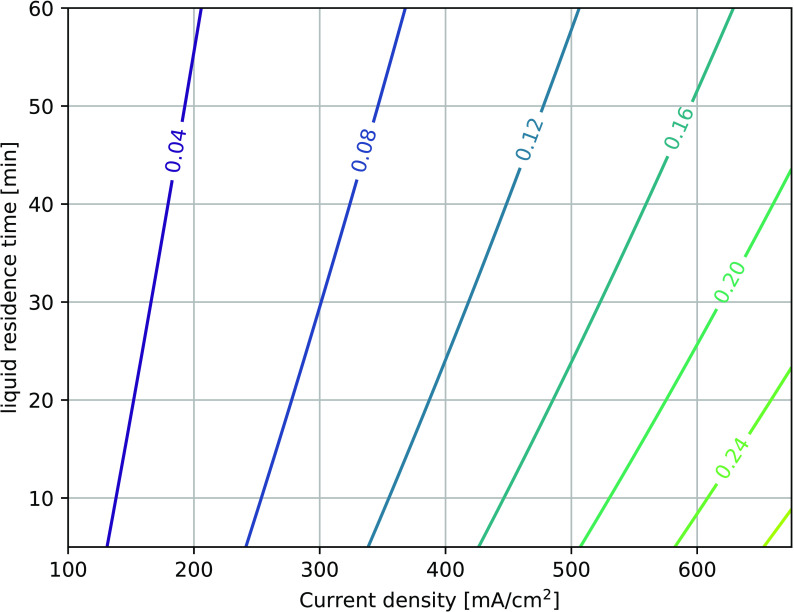
Effect of liquid residence time and current density on peroxodicarbonate concentrations. Iso lines with constant peroxodicarbonate concentration in M. Simulation conditions: 1 M Na_2_CO_3_ feed concentration, *T* = 11 °C, circulation flow rate of 14 L/min.

The validated model is used to explore how the design parameters affect the steady-state performance of the peroxodicarbonate reactor setup. The effects of the electrolysis temperature, the liquid residence time, and the current density with respect to concentration of produced oxidizer and the specific energy consumption (SEC) are investigated. From eq 3 it is known that an increased temperature increases the degradation rate of peroxodicarbonate. This leads to a reduced peroxodicarbonate concentration. With the cell design used, the model predicts a drop of approximately 36 mM, when increasing the production temperature to 10 °C. The effect of current density and liquid residence time on the peroxodicarbonate concentration is displayed in Figure 7. The concentration is depicted using iso line (= constant) concentrations.

The current density for a fixed electrode area will represent the total current charge. As expected, the concentration of peroxodicarbonate increased with increasing current density. If the residence time of the liquid in the system is increased, then there is more time for the decomposition reaction to take place. Thus, the peroxodicarbonate concentration decreases with an increasing liquid residence time.

The electrochemical production of concentrated aqueous peroxodicarbonate solutions has recently been successfully demonstrated by Seitz et al.^45^ Concentrations of close to 1 M peroxodicarbonate are achievable when operating with boron-doped diamond anodes, similar to those used in this work. The main difference is that Seitz et al. operated with significantly higher current densities of 3.33 A/cm^2^ and with specially designed cooling circuits. In addition, a multicomponent electrolyte was applied.

##### SEC as a Key Performance Parameter

**Figure 8:**
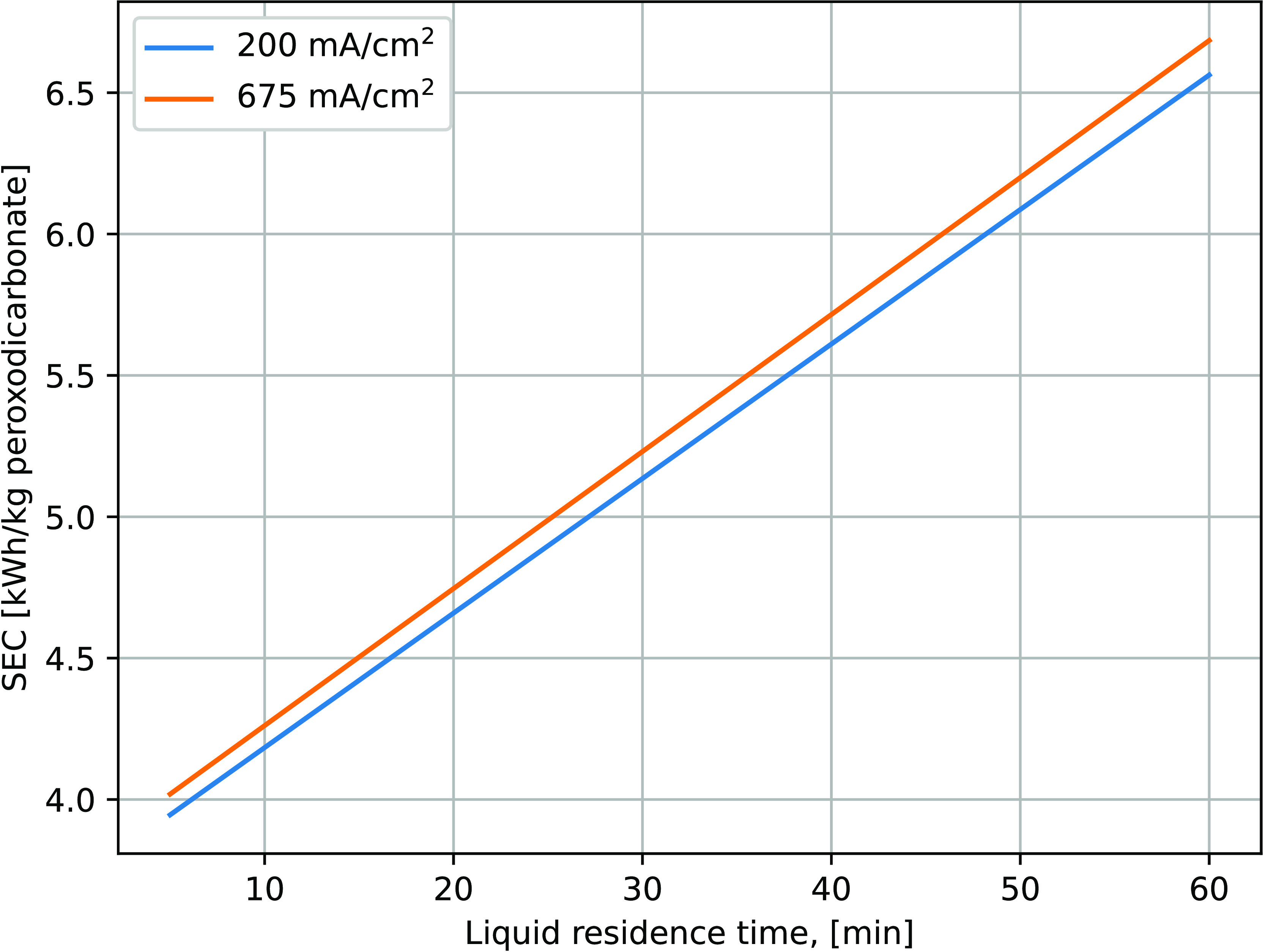
Effect of liquid residence time and current density on SEC [kWh/kg peroxodicarbonate]. Simulation conditions: 1 M Na_2_CO_3_ feed concentration, *T* = 11 °C, circulation flow rate 14 L/min.

SEC refers to the amount of energy used to produce a unit of product, here peroxodicarbonate, and can be calculated by using eq 5. It is a critical metric in various industries, especially when assessing energy efficiency, and is used as a key performance parameter for the peroxodicarbonate reactor setup used.
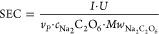
5In addition to the concentration of peroxodicarbonate (*c*_Na_2_C_2_O_6__) in mol/L, the applied electrical current (*I*) in *A*, the product rate (*v*_*P*_) in L/h, the molar mass of peroxodicarbonate (*Mw*_Na_2_C_2_O_6__) in g/mol, and the cell voltage (*U*) in V are taken into account in this formula. The latter is estimated by an empirical correlation from Chardon et al.^42^ and extended to take into account the electrode distance and the electrolyte conductivity as described by Ziogas et al.^44^ The electrolyte conductivity is affected by the formation of hydrogen gas in eq 1 and is based on a relation proposed by Bruggemann.^51^ The voltage as a function of the potentials of the electrodes and ohmic voltage drop does have a significant impact on the SEC. Typical voltages that were used in the pilot are between 4.87 and 6.75 V. It is noteworthy that SEC displays the electrical power for a given product rate, whereas the Faraday efficiency is a measure of how efficiently the amount of charge is converted to a product. In this study the concentration of peroxodicarbonate is measured experimentally. In eq 5, this experimental concentration could be replaced by the theoretically possible concentration of peroxodicarbonate multiplied by the current efficiency. The theoretical concentration of peroxodicarbonate from 1 M sodium carbonate is 0.5 M. For a measured concentration of 0.2 M the Faraday efficiency would be 40%. As displayed in Figure 8, increasing the liquid residence time significantly increases the specific electricity consumption, whereas the current density over the cell (for a constant value of the liquid residence time) does not have a particular effect. The reduction in specific electrical consumption with reduced liquid residence time is easily explained when considering the thermal decomposition reaction that takes place within the liquid holdup volume, as expressed in eq 4. Thus, to minimize specific energy consumption, the liquid residence time in the circulation loop around the electrochemical reaction should be minimized.

Waldvogel and coworkers report an energy consumption of 0.65 kWh/mol for the production of 0.337 M peroxodicarbonate,^45^ whereas Chardon et al. used 2.6 kWh/mol for 0.28 M.^42^ Converting the SEC data displayed in Figure 8 (4–6.4 kWh/kg oxidizer) into kWh/mol oxidizer, the values are in the same order of magnitude (0.66–1.1 kWh/mol oxidizing agent) as the literature data. If the reservoir in the pilot plant had been smaller, resulting in a residence time of about 5 min, the energy consumption achieved in this case would have been almost the same as what was reported by Seitz et al. They reported a decrease in Faraday efficiency with higher peroxodicarbonate concentrations up to 1 M. For the SEC peroxodicarbonate concentrations of 0.87 and 0.919 M, for example, Seitz et al. calculated an energy consumption of 16 and 25 kWh/kg peroxodicarbonate, respectively. In this work, the effects of a continuous feed and product flow were also taken into account. Remarkably, both Seitz et al. and Chardon et al. produced peroxodicarbonate continuously by circulating a fixed volume of carbonate solution. In this work, the effects of continuous feed and product flow were considered additionally.

### Performance of the Pilot Plant

In the reactor product, 22 different aromatic compounds were identified using the National Institute Standard and Technology database.^52^ In addition to the target monomer vanillin, similar structures such as acetophenone, p-cresol, acetovanillone, and guaiacol could be detected as byproducts of the conversion.

**Figure 9:**
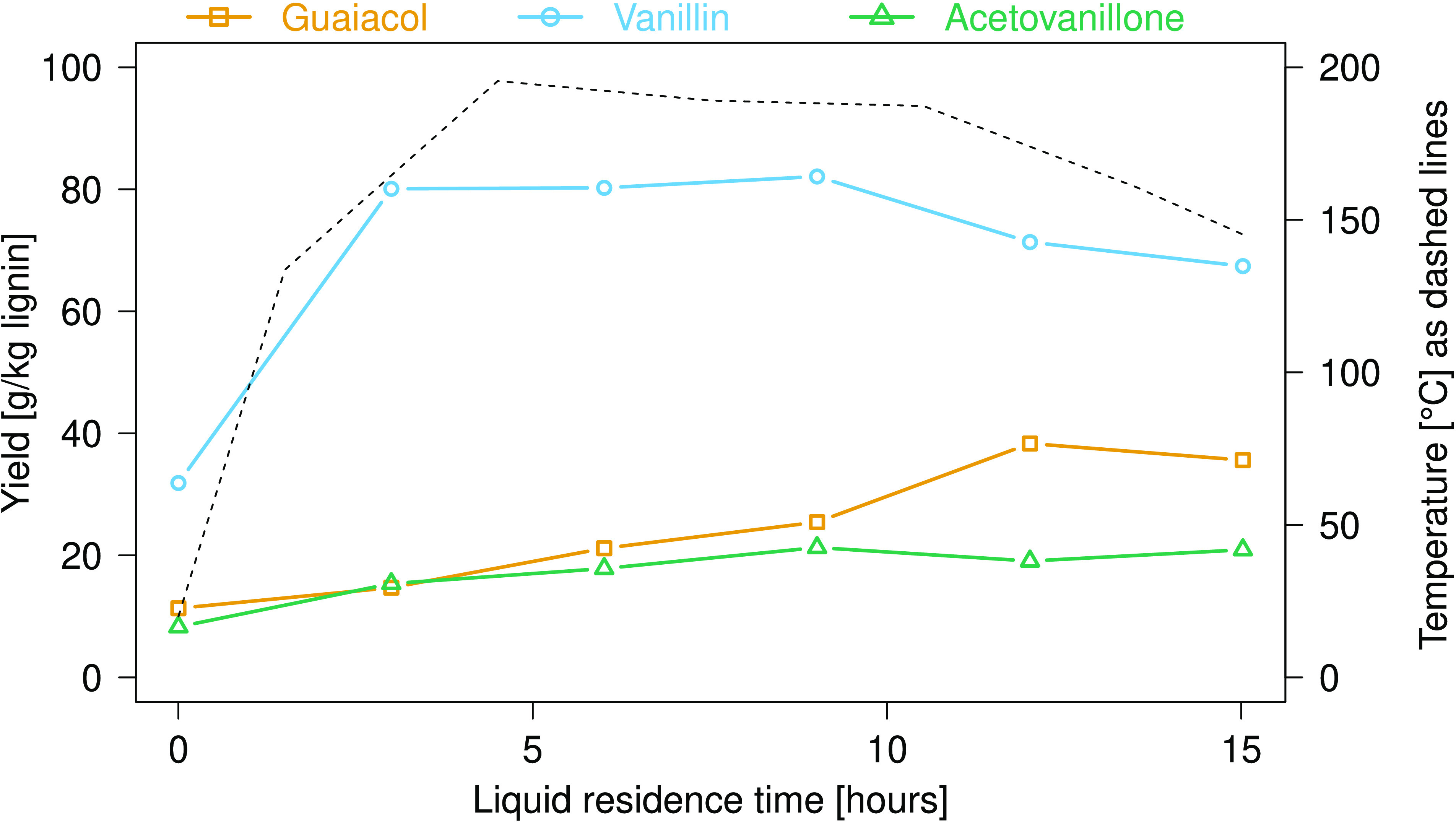
Yield evolution of different phenolic monomers along the reactor/over time. Results were measured by GC-MS. Experimental parameters: 0.1 wt % Kraft lignin in 3 M NaOH solution with a peroxodicarbonate to lignin ratio of 3.6 kg/kg. The temperature profile is indicated by dashed lines.

The yields quantified by GC-MS are displayed in Figure 9. With a yield of up to 8 wt %, vanillin is formed reasonably selectively compared to other phenolic monomers. The yield of guaiacol appears to increase with longer reaction times, while that of acetovanillon remains unchanged regardless of the sampling point. It should be noted that the maximum vanillin yield is already achieved at the second sampling point, after approximately 3 h residence in the heated plug flow reactor at a temperature of 150 °C. Both the liquid residence time and the temperature have a lower value compared to what Zirbes et al.^31^ reported to receive a similar yield. Once formed, vanillin remains stable for several hours. The decrease in the yield of vanillin and the simultaneous appearance of other moieties indicate a relocation of methyl groups and other side chains on the phenolic ring. The selectivity of this process with respect to vanillin observed in these experiments and yields confirms the observations of Zirbes et al. and shows a successful scale-up of this approach.

**Figure 10:**
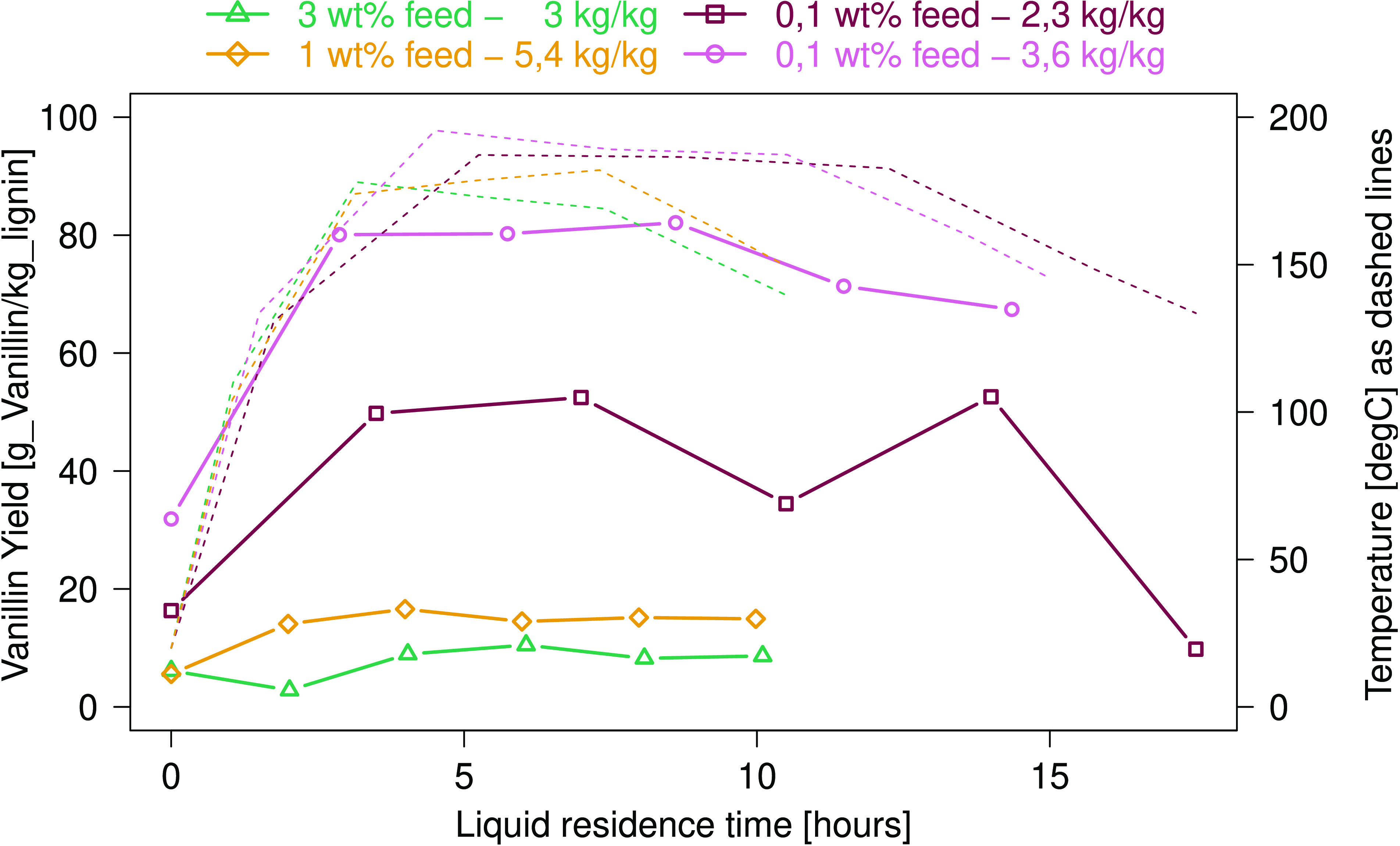
Effect of the lignin concentration in the feed solution (3 M NaOH) and the peroxodicarbonate to lignin ratio (kg/kg) on the vanillin yield. The temperature profile is indicated in dashed lines.

To investigate the effect of peroxodicarbonate on the oxidative degradation of Kraft lignin to vanillin, we ran a reference experiment without peroxodicarbonate and only sodium carbonate solution instead. This clearly demonstrated that thermolysis alone without applying peroxodicarbonate does not achieve the same yields as expected. Impressively, the vanillin yield can be increased by a factor of 4 using peroxodicarbonate under similar experimental conditions in the plug flow reactor. Using a full factorial design, Zirbes et al.^31^ identified the temperature and heating time of the thermolysis as crucial parameters. The amount of oxidizer was categorized as marginal but necessary for efficient depolymerization to vanillin. As the lignin concentration and the concentration of the subsequent reactor product are also of great importance for the downstream separation and overall efficiency of the process plant, various feed concentrations were tested (Figure 10). It is evident that the yields are directly dependent on the lignin concentration used. When the ratio of peroxodicarbonate to lignin is maintained or even increased, the concentration of the lignin feed cannot be increased without compromising yields. Zirbes et al. described one experiment with increased lignin concentration by a factor of 5 (corresponds to 1 wt %), which resulted in a similar vanillin yield of 5 wt % compared to earlier experiments. These findings could not be confirmed in the scale-up, as the experiments of this work using the same conditions only resulted in lower yields of approximately 2 wt %. In this study, the focus was put on the conversion of Kraft lignin. However, the impact of the lignin source will be examined in future investigations.

The lignin concentration is significantly reduced by the addition of a peroxodicarbonate solution. This dilution can be expressed by a simple mass balance around the mixing point between lignin and peroxodicarbonate eq 6
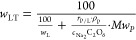
6where *w*_LT_ is the weight fraction of lignin and its depolymerization products in the thermal depolymerization reactor; *w*_L_ is the weight fraction of lignin in the feed; *r*_P/L_ is the peroxodicarbonate to lignin ratio (mass basis); and ρ_P_ and *Mw*_*P*_ are peroxodicarbonate density and molecular weight, respectively.

**Figure 11:**
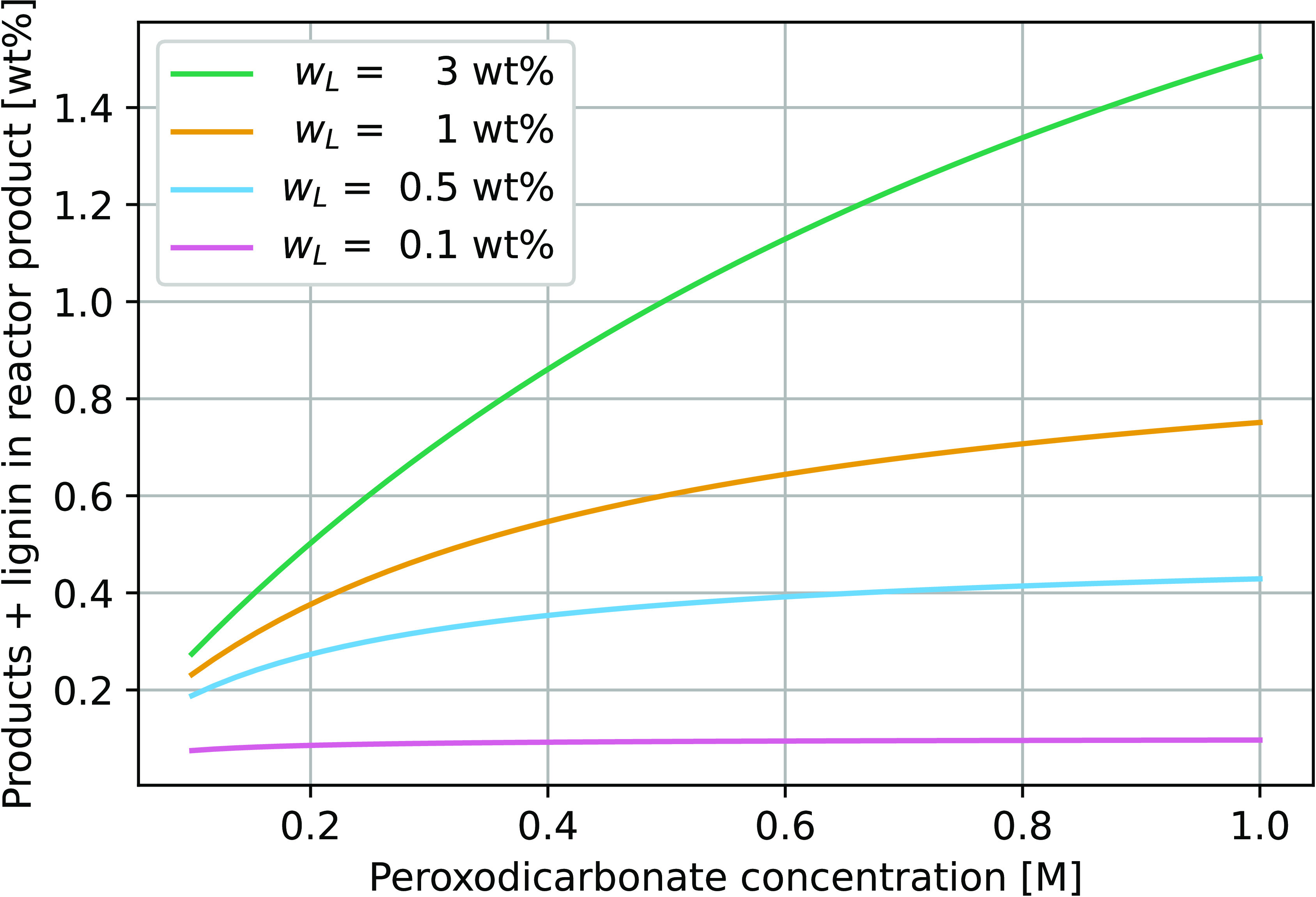
Effect of lignin feed concentrations (*w*_L_) dissolved in a NaOH solution. Concentration of lignin and its depolymerization products in the reactor product as a function of the peroxodicarbonate concentration. The weight-based ratio of oxidizer to lignin was 5 in all cases.

Figure 11 illustrates the dilution effect of lignin and its depolymerization products in the thermal reactor, according to eq 6. Assuming a fixed ratio of oxidizer to lignin, the variance of the oxidizer has an unambiguous effect on the product concentration, especially for higher lignin feed and oxidizer concentrations.

There is an undeniable potential of working with higher concentrations of lignin and thus increasing the overall concentration of target compounds (e.g., vanillin) in the product stream which would make the downstream process more efficient and less energy-intensive. However, this will only be economically viable if the vanillin yield achieved from lower lignin concentrations will also be feasible for higher lignin concentrations. The total vanillin concentrations in the outlet of the experiments conducted in this study were approximately 0.03 wt % for a 3 wt % lignin feed and 0.008 wt % for the 0.1 wt % case, both achieved with a peroxodicarbonate concentration of 0.2 M peroxodicarbonate. Within this study it was not tested how high lignin concentrations can be increased when applying high concentrations of peroxodicarbonate to achieve good vanillin yields. In an accompanying study of the technical–economic profitability and the life cycle assessment of this lignin depolymerization process, the high dilution rate and the production of peroxodicarbonate were identified as the main bottlenecks. It is reported that the electrochemical process can barely be profitable under the investigated conditions due to the high costs (CAPEX and OPEX of 13.76 and 5.91 kg of lignin, respectively). Furthermore, they reported the carbon footprint of the pilot plant for producing 1 kg of vanillin to be 1.37–1.43 kg CO_2_ equivalents. Cabeza Sánchez et al. further compared this value with the LCA results of the existing processes from Borregaard and concluded that the emissions are very similar.^53^ Looking at the impact categories for the new electrochemical process, they report that the production of peroxodicarbonate scored highest in 4 of 6 environmental impact categories and second highest in the human toxicity environmental impact category.^53^ By integration of the techno-economic analysis, informed decisions can be made for further process optimization.

## Conclusion

In conclusion, the synthesis of peroxodicarbonate at the pilot scale was successfully carried out. Data obtained from this process served as the basis for a model encompassing both steady-state operation and transient behavior. Exploration of various design parameters, including production temperature, liquid residence time, and current density, and their impact on peroxodicarbonate concentration and specific energy consumption was facilitated by the validated model. Optimization of operating temperature and the peroxodicarbonate circulation loop will be determined by considering the cost of refrigeration or cooling water utilities and the loss of peroxodicarbonate due to thermal degradation. Notably, the specific energy consumption increases with a higher peroxodicarbonate concentration, indicating an economic optimum for peroxodicarbonate. Additionally, the oxidative degradation of Kraft lignin using peroxodicarbonate as a “green” oxidizer was successfully demonstrated at TRL 6. Remarkably, vanillin yields of up to 8 wt % were achieved. However, the fact that the dilution effect has a detrimental effect on the economic sustainability of this process should not be overlooked.

## Materials and Methods

The experimental details, analysis techniques, and additional information are provided in the Supporting Information.

### Experimental Setups

**Figure 12:**
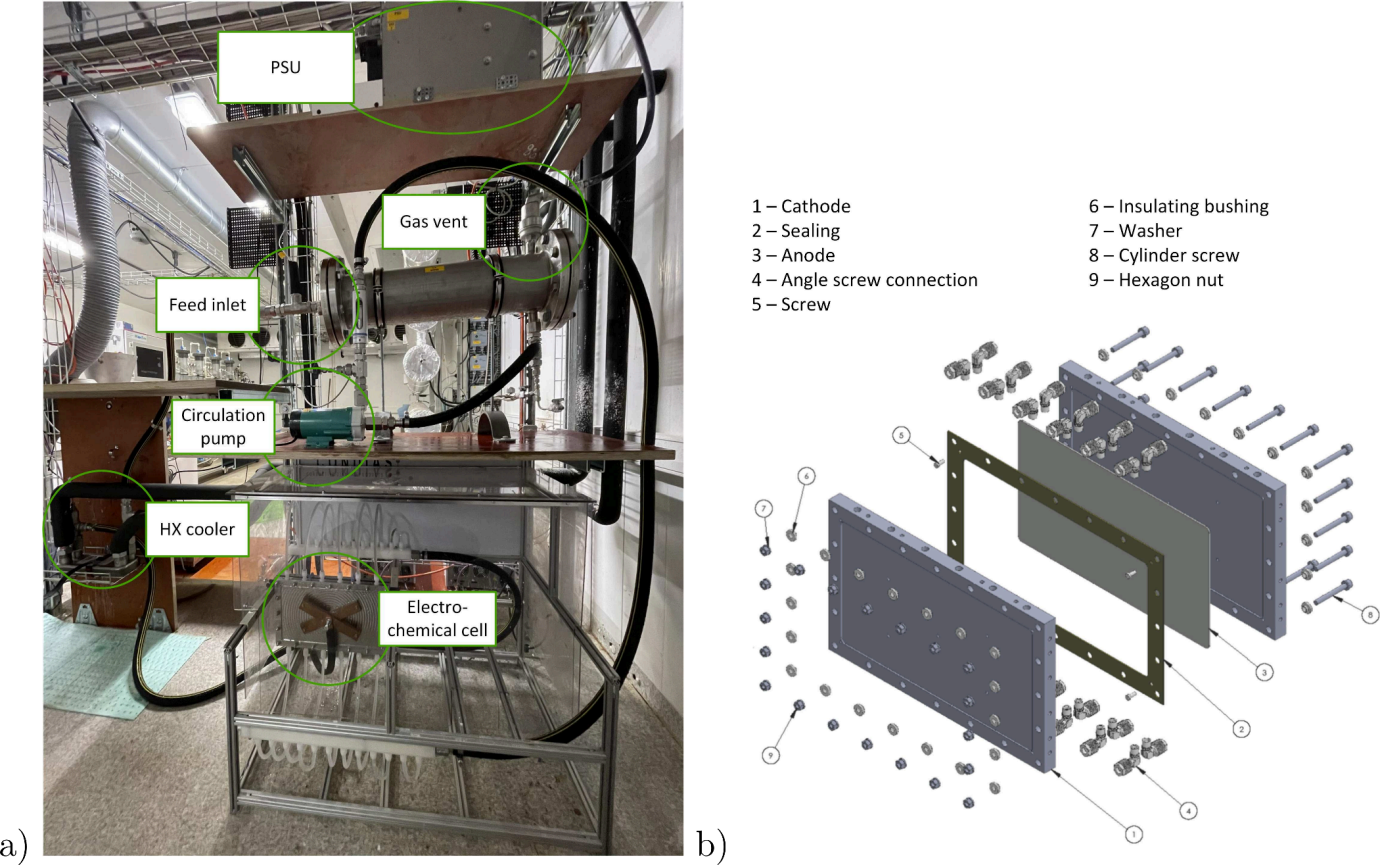
Peroxodicarbonate reactor setup. (a) An overview with the main components indicated and (b) electrolysis cell by CONDIAS GmbH.

**Figure 13:**
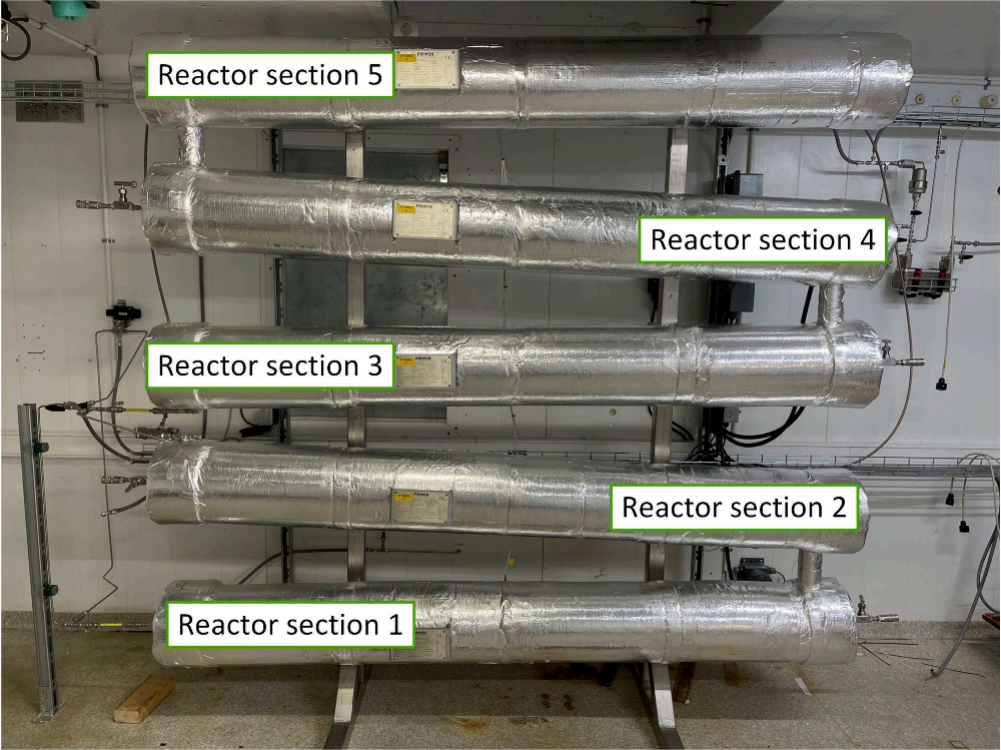
For the thermal depolymerization reactor, two independently controlled temperature zones are installed. Inline temperature transmitters are located in the center of each reactor section. Sample points are located at the end of each reactor section.

Figure 12 shows the physical realization of the electrolyzer for the production of peroxodicarbonate described previously in Figure 4. Figure 13 shows the thermal depolymerization reactor where peroxodicarbonate is mixed with lignin and allowed to undergo thermal depolymerization in a plug flow reactor.

**Figure 14:**
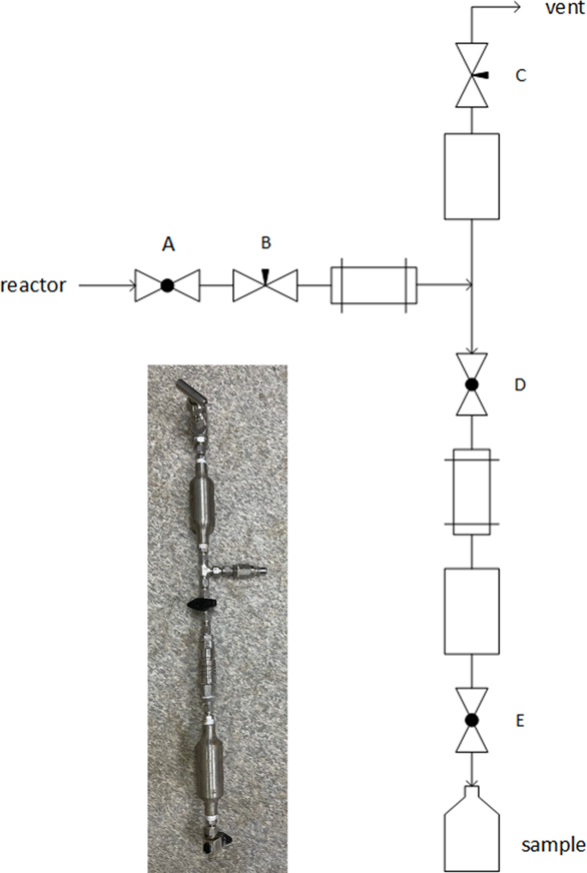
Custom-made device for sampling along the reactor.

The sampling device (Figure 14) is an in-house design to facilitate taking samples while the pressurized process is in operation. A sample of 60 mL is taken under the operating pressure of the reactor (16 bar). After the device has been disconnected from the reactor and cooled, the apparatus can be vented, and the sample can be drained prior to analysis. Reactor samples were gathered typically 18 h after a reasonable steady state temperature profile has been established in the sections of the thermal depolymerization reactor. Steady state was typically reached after 8–12 h upon start.

### Modeling

The reaction mechanism taking place at the anode is
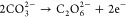
7The reaction mechanism taking place at the cathode is
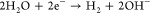
8Chardon et al.^42^ measured the instantaneous current efficiency as a function of current density. The results indicate a linear relationship where *k*_η_ and *m*_η_ are empirical constants.
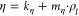
9The cell voltage is estimated by an empirical correlation from Chardon et al.^42^ and extended to take into account the electrode distance and the electrolyte conductivity as described by Ziogas et al.^44^
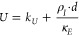
10The parameter *k*_*U*_ is the constant in the linear correlation for the cell voltage. The electrolyte conductivity κ_*E*_ is affected by the formation of hydrogen gas in eq 1. Bruggemann^51^ proposed the following relation:
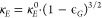
11

#### Steady State

A steady-state model may be formulated from a mass balance around the electrochemical cell and the reservoir. The difference in Na_2_C_2_O_6_ flow rate in the feed and product stream is equal to the production rate of Na_2_C_2_O_6_ formulated in eq 1.
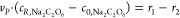
12Note that we need to add a practical constraint when solving eq 12 to ensure the production rate of Na_2_C_2_O_6_ does not exceed the available amount of Na_2_CO_3_ being fed into the reactor system, i.e.,
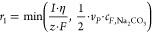
13Since the feed tank only contains aqueous Na_2_CO_3_eq 12 can be reformulated to express the peroxodicarbonate product concentration *c*_*R*_ as a function of production rate.
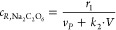
14The total (dry) gas production from the electrochemical cell is
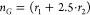
15The concentration of hydrogen, carbon dioxide, and oxygen in dry gas is expressed as
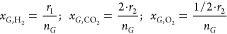
16The concentration of the other reaction products at the steady state is derived from the mass balance around the reactor system:
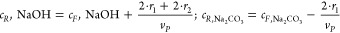
17Model parameters were fitted by solving the following least-squares optimization problem:
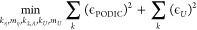
18where  and .

#### Dynamic Model

The transient behavior of the product concentration in a single reservoir reactor setup may be formulated by taking into account the feed and product stream:
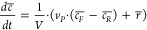
19where liquid phase concentration and reaction rates are defined according to the stoichiometric reactions in eqs 1 and 3:
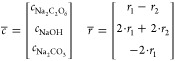
20The discontinuous min operator in eq 13 will introduce numerical problems once full conversion of sodium carbonate is achieved. In order to mitigate this problem, the min operator is replaced by a sigmoidal function which ensures that *r*_1_ is close to zero as the Na_2_C_2_O_6_ concentration approaches zero. The sigmoidal function has the form:
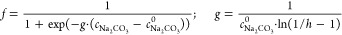
21The expression for the Na_2_C_2_O_6_ formation rate is reformulated for the dynamic model as a combination of eqs 2 and 21 into:
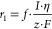
22

## Supporting Information Available

General information on the experimental setups, details on the used chemicals, the characterization of peroxodicarbonate, analysis methods, and further information on the modeling (PDF)
