# Supplementary material for: Pilot Scale Electrolysis of Peroxodicarbonate as an Oxidizer for Lignin Valorization
Source: ACS Sustain Chem Eng. 2024 Jul 18;12(30):11283–96. doi: 10.1021/acssuschemeng.4c02898 (PMC11289759; doi:10.1021/acssuschemeng.4c02898)
Supplement: Supplementary file 1 — sc4c02898_si_001.pdf [file sc4c02898_si_001.pdf]

Supporting Information

Pilot scale electrolysis of peroxodicarbonate as an oxidizer for lignin valorization

Theresa Rücker,[†,§] Torbjørn Pettersen,[†] Hannah Graute,[‡] Bernd Wittgens,[†] Tobias Graßl,[¶] and Siegfried R. Waldvogel[*,‡,§]

†*Process Technology, SINTEF Industry, Trondheim, Trøndelag, NO-7465, Norway*

‡*Karlsruhe Institute of Technology, Kaiserstraße 12, 76131 Karlsruhe, Germany*

¶*CONDIAS GmbH, Fraunhofer Straße 1b, 25524 Itzehoe, Germany*

§*Max Planck Institute for Chemical Energy Conversion, Stiftstraße 34-36, 45470 Mülheim an der Ruhr, Germany*

E-mail: siegfried.waldvogel@cec.mpg.de

Number of pages: 30

Number of figures: 6

Number of tables: 9

Contents

1 List of symbols, superscripts and abbreviations **3**

2 Setups **5**

2.1 Process control system 7

3 Materials **9**

4 Characterisation of peroxodicarbonate **10**

4.1 DFT calculations 10

4.2 ELF calculations 11

4.3 ORCA scripts 12

5 Analysis **18**

5.1 Peroxodicarbonate 18

5.2 Components and their yields derived from lignin 19

5.2.1 Work-up procedure 19

5.2.2 GS-MS screening and quantification 19

6 Modelling **22**

References **24**

1 List of symbols, superscripts and abbreviations

Table S1: List of symbols.

Symbol	Description	Unit
A	frequency factor for rate constant	1/s
a	electrode area	cm^2
c	molar concentration	M = mol/l
$C(\vec{r})$	positional dependent parameter	
E	activation energy	J/mol
F	Faradays constant	96485 C/mol
I	cell current	A
k	rate constant	1/s
m	mass flow rate	kg/s
Mw	molecular weight	g/mol
n	molar flow rate	mol/s
n_i	occupational number	-
$\bar{N}$	electron population	-
P	pair probability distribution	-
r	reaction rate	mol/s
R	gas constant	8.314 J/(mol*K)
U	terminal cell voltage	V
V	liquid volume	l
v	volumetric flow rate	l/s
x	mole fraction	-
z	amount of charge applied per peroxodicarbonate formed	2
η	current efficiency	-
ϵ	relative deviation between model prediction and experimental data	-
Ω	basin	-
Φ	orbital	-
ρ	electron spin density	
ρ_I	current density ($mA/cm^2 = 10\ A/m^2$)	A/m^2
ξ	conversion of a component	-

Table S2: List of subscripts.

Subscript	Description
0	feed stream
1	electrochemical production of peroxodicarbonate
2	decomposition of peroxodicarbonate
F	Na_2CO_3 feed stream to reservoir
G	gas vent stream from reservoir
h	homogeneous electron gas
k	experimental data point index
P	product stream from reservoir
R	reservoir

Table S3: List of abbreviations.

Abbreviation	Description
CPCM	conductor-like polarizable continuum model
DFT	Density functional theory
ELF	electron localization function
LP	lone pair
P&ID	Piping and instrumentation diagram
TRL	Technology Readiness Level

2 Setups

The pilot plant (Figure S1) was installed at the multi-phase flow laboratory at SINTEF in Trondheim. It consists of a feed system for raw materials, an electrochemical reactor setup for continuous production of peroxodicarbonate, and a thermal depolymerisation reactor for oxidation and thermal depolymerisation of lignin. The reactor system is designed for continuous operation, while reactor product separation and upgrading is performed in a semi-batch separation process.

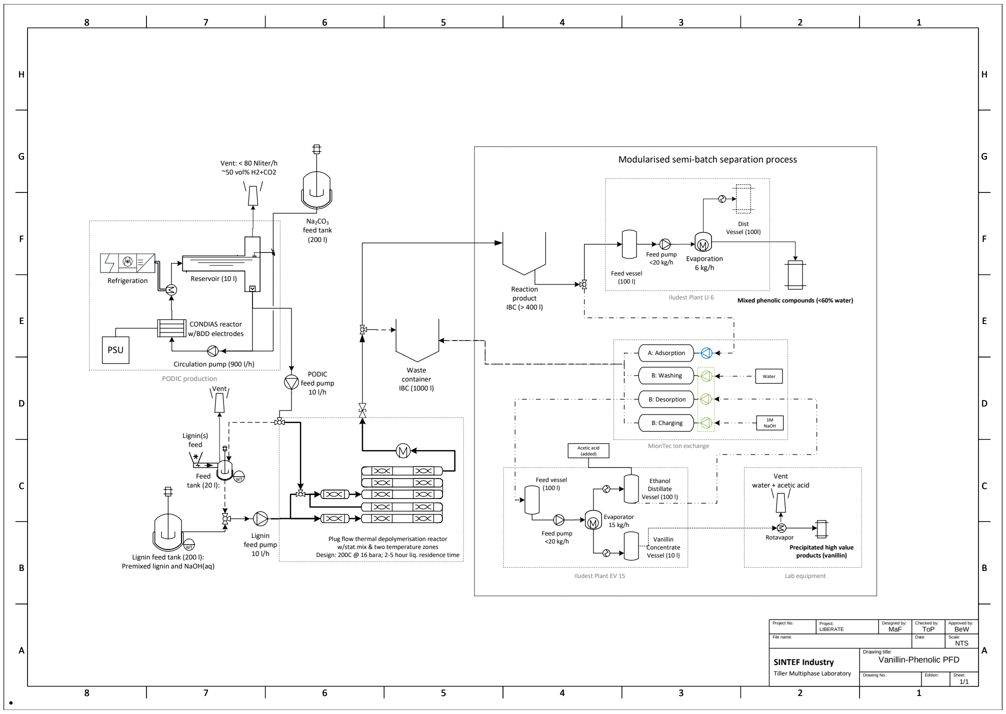

Figure S1: Process flow diagram of the electrochemical lignin depolymerization plant.

The peroxodicarbonate reactor setup (Figure S2) was built based on the following Piping and instrumentation diagram (P&ID):

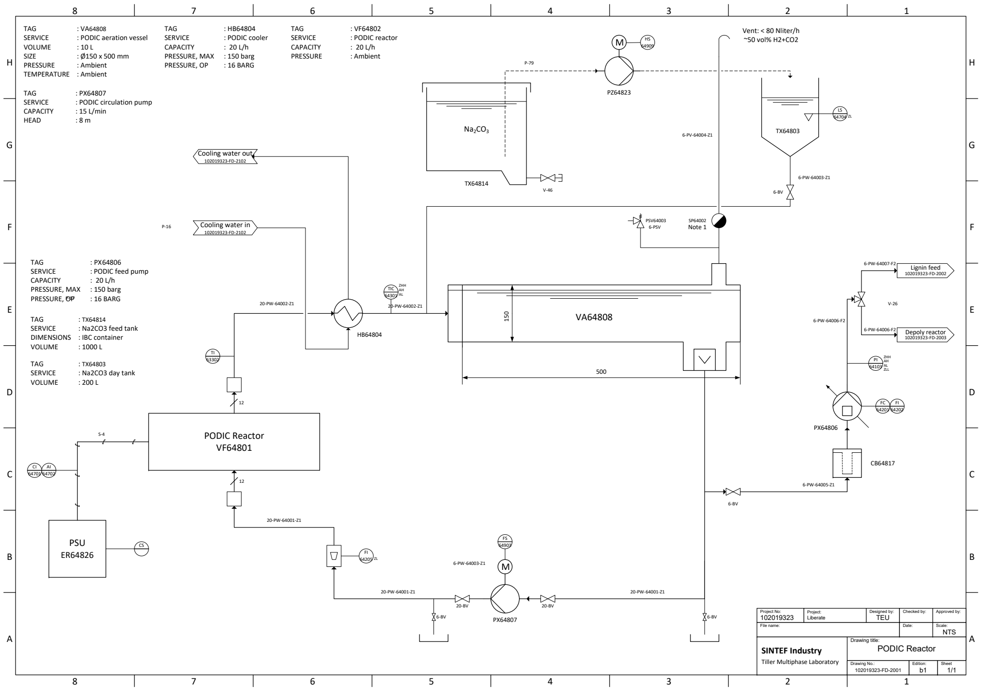

Figure S2: P&ID of the electrochemical reactor configuration with internal recirculation loop and feed and bleed reservoir.

The electrochemical cell is powered by a power supply manufactured by plating electronic GmbH. The power station pe4606 provides up to 300 A at up to 10 V with a maximum net effect of 2800 W. Cooling downstream of the electrochemical cell was provided by a fusion-bonded plate heat exchanger, type 14-30H, manufactured by Alfa Laval Nordic AS. The cooling capacity was 3 kW with a minimum temperature approach of 3 ^{o}C. The cooling water temperature was kept above 8 ^{o}C to avoid the precipitation of sodium carbonate. The circulation pump was a model MD-30RZM-2020N from Iwaki Co., Ltd. with a magnetic drive. The maximum capacity was 15 L/min. Noteworthily, that all data were recorded over more than a year, without significant change in the performance of the cell. Key design parameters are displayed in Table S4.

Table S4: Key design parameters for the pilot plant depicted.

Properties	Description	Value
Anode	Boron-doped diamond	435 cm^2
Cathode	Stain-less steel	-
Inter-electrode gap	Anode - cathode	1.5 mm
Anode dimensions	Width x height	30 cm x 14,5 cm
Current density	Operating range	200-695 mA/cm^2
Liquid holdup	Gas-liquid separator	10 L
	Electrolysis cell	65 mL

The thermal depolymerization reactor (Figure S3) was built based on the following concept:

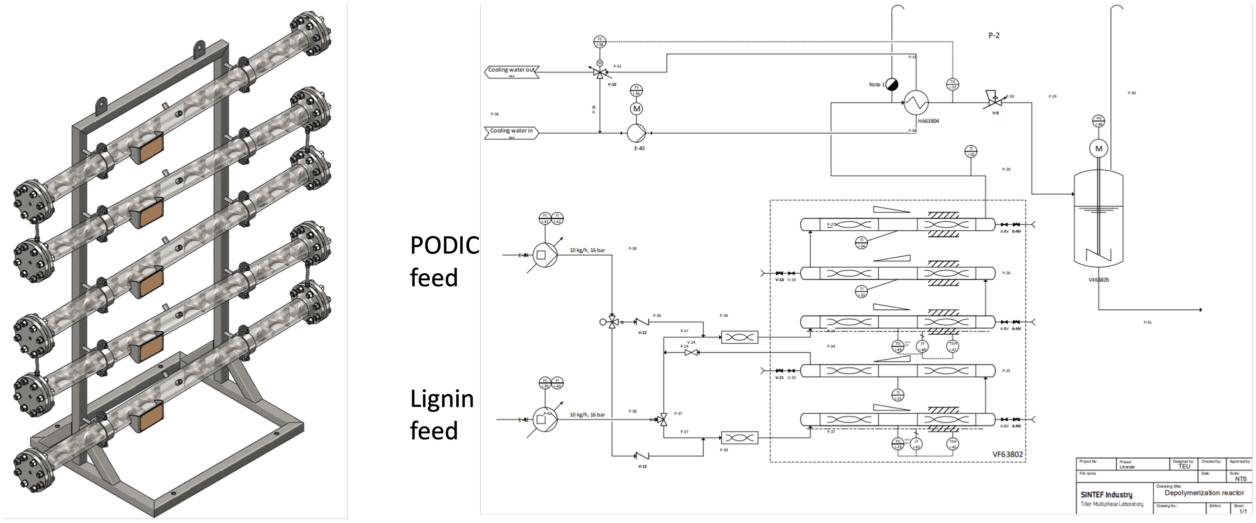

Figure S3: Thermal depolymerisation reactor design as a plug flow reactor with internal static mixers for radial mixing. Feed locations are at the entrance of reactor section 1 and 3.

2.1 Process control system

A process control system (Implemented by VisonTech AS in IGSS from Schneider Electronics) has been established for the lignin feed and reactor systems in the pilot which enable 24 hours unmanned operation of the unit. The control system is linked to a Labview based datalogger service which stores all process data to a SQL database which is part of the lab infrastructure.

The human machine interface (HMI) for the peroxodicarbonate and the thermal depolymeri-

sation reactor are shown in Figure S4 and S5.

Control parameters for peroxodicarbonate reactor (electrolyzer) are:

- Start/stop signal to circulation pump PX64807
- Start/stop of the power supply unit (PSU)
- Set-point for output current from the PSU – given as current density
- Set-point for temperature control of the circulation loop via TIC64301
- Start/stop of the peroxodicarbonate feed pump PX64806
- Set-point for volumetric feed flow rate from PX64806 [ml/min]

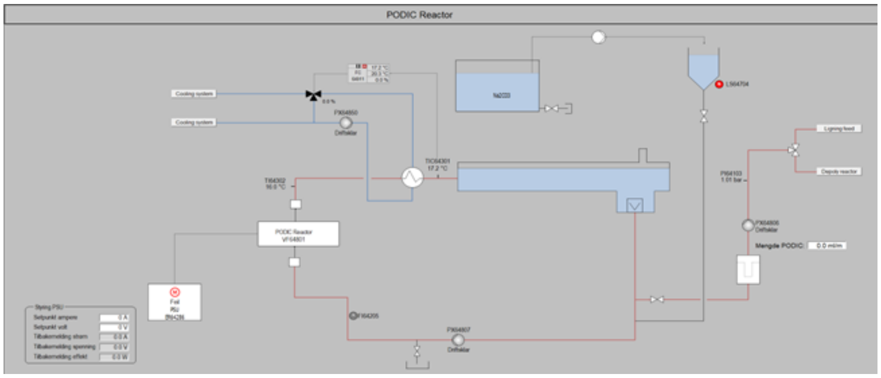

Figure S4: Process control HMI for peroxodicarbonate reactor system.

Input parameters are:

- Distribution of peroxodicarbonate feed to either reactor section 1 or 3 via FC64913
- Percent of time period where peroxodicarbonate goes towards reactor section 3 (default is zero)
- Length of time period (default 20 min)
- Heat input to heating zone 1, 0-1900 W
- Heat input to heating zone 2, 0-1900 W

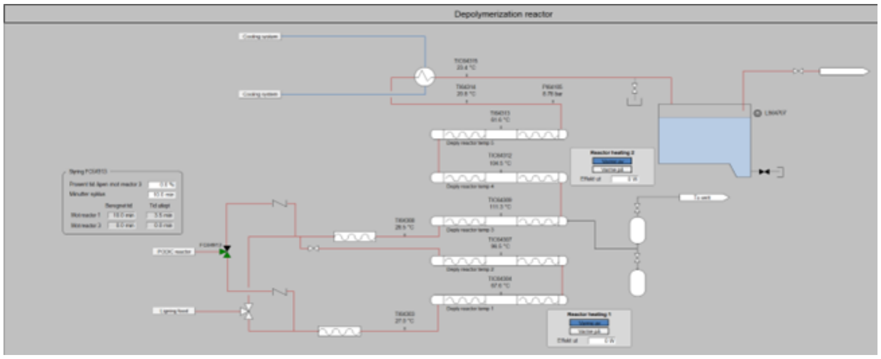

Figure S5: Process control HMI for thermal polymerisation reactor.

3 Materials

The Kraft lignin used was the Lineo® Classic product from Stora Enso. It is a technical lignin derived from spruce and pine and was isolated by means of the LignoBoost process at the Sunila mill, Kotka, Finland.

Sodium carbonate for the electrochemical production of peroxodicarbonate was industrial grade calcinated and granulated soda purchased from Solberg Industri AS. See Table S5 chemical composition and physical data. The soda was delivered in 25 kg bags and mixed with tap water in a 1000 l IBC container with stirring.

Table S5: Chemical and physical data for sodium carbonate.

**Chemical data**	**Specification**	**Typical values**	**Unit**
Sodium carbonate, Na_2CO_3	>= 99.6	99.6 – 99.8	%
Sodium chloride, NaCl	<= 0.25	0.09 – 0.12	%
Iron oxide, Fe_2O_3	<= 0.002	0.0002 – 0.001	%
Sodium sulphate, Na_2SO_4	<= 0.02	< 0.02	%
Insoluble in water	<= 0.02	0.007 – 0.009	%
**Physical data**			
Loss during heating (70 → 300 °C)	<= 0.5	0.1 – 0.2	%
Bulk density	0.95 – 1.2	0.97 – 1.1	kg / dm^3
Particle distribution			
> 2.0 mm	<= 0.5	0 – 0.2	%
> 1.6 mm	<= 1	0 – 0.5	%
< 0.1 mm	<= 5	0.1 – 1.0	%

Aqueous sodium hydroxide for the dissolved lignin feed stream was purchased from Solberg Industri AS at 46 wt% concentration and was stored in an isolated (at 30 ^oC) IBC container, prior to mixing with water and lignin in the 200 l feed tank. See Table S6 for chemical and physical data.

Table S6: Chemical and physical data for aqueous sodium hydroxide.

**Chemical data**	**Specification**	**Typical values**	**Unit**
Sodium hydroxide, NaOH	49.0 – 51.0		%
Sodium chloride, NaCl	< 250		ppm w/w
Sodium carbonate, Na_2CO_3	< 1000		ppm w/w
Sodium sulphate, Na_2SO_4	< 100		ppm w/w
Sodium chlorate, $NaClO_3$	< 60		ppm w/w
Iron, Fe	< 5		ppm w/w
**Physical data**			
Specific density (50%, 20 ^oC)		1.52	g/cm^3
Freeze point		12	^oC

4 Characterisation of peroxodicarbonate

4.1 DFT calculations

The DFT calculations have been performed with the ORCA program package[1] in version 4.2.1 (x86-64, Intel Core i7-1255U/12 cores). The def2 basis sets, especially def2-TZVP and def2-TZVPP (def2/JK) for the anionic species have been used and B3LYP was selected as hybrid functional. If necessary, the BP86 functional was used for pre-optimizations.[2–14]

For all calculations, the conductor-like polarizable continuum model (CPCM) was used with water as solvent, which is implemented in ORCA. The anionic geometries and atom coordinates have been setup based on common bond angles and bond length. The geometry was then optimized using iterative single-point energy calculations.

4.2 ELF calculations

Based on the results of the DFT calculation, the ELF was calculated. The ELF offers a method to localize paired electrons in chemical systems and hence, it strongly correlates with the chemical intuitive concept of bonds and lone pairs. The pair probability distribution (P) to find an electron at a given spacial position ($\vec{r}$) with the same spin as the probe at a given distance (s) will be zero if $s = 0$ due to the Pauli principle:[15–27]

$$P(\vec{r}, s = 0) = 0$$

If P is expanded to a power series and under the assumption that s is small, a positional dependent parameter C($\vec{r}$) is analyzed. Within the Hartree-Fock theory and the consideration of the Salater determinant as the simplest form of a wave function, C($\vec{r}$) can be described using the occupation number (n_i), the orbital (Φ) and the electron spin density (ρ).

$$C(\vec{r}) = \frac{1}{2}\sum_i n_i |\nabla\phi_i|^2 - \frac{1}{8}\frac{|\nabla\rho|^2}{\rho}$$

For practical reasons, Becke and Edgecombe[17] introduced the homogeneous electron gas (h) as a reference system on the one hand

$$C(\vec{r}) \rightarrow C_h(\vec{r}) \sim \rho^{5/3}$$

and a scaling of the function to a range between [0..1] to be able to compare results from different calculations:

$$\mathrm{ELF} = \frac{1}{1 + \left(\frac{C(\vec{r})}{C_h(\rho(\vec{r}))}\right)^2}$$

The ELF value converges to 1 at regions where the probability to find paired electrons is very high (e.g. paired electrons, lone pairs) and will be 0.5 in a homogeneous electron gas. Usually, the ELF will be analyzed in a topological manner, where local maxima of the functions are called attractors and spacial regions are created by gradient calculations in the ELF where such a region is called ELF-basin (Ω). The number of electrons ($\bar{N}$) populating such a spacial region is calculated by integrating the electron density in the ELF-Basins.

$$\bar{N}(\Omega) = \int_{\Omega} \rho(\vec{r})\, \mathrm{d}\vec{r}$$

ELF calculations, topological analysis and electron density integration were performed with Multiwfn.[28]

4.3 ORCA scripts

```
Peroxodicarbonate

               *****************************************************
               *                     SUCCESS                       *
               *           SCF CONVERGED AFTER   5 CYCLES          *
               *****************************************************

Setting up the final grid:

General Integration Accuracy     IntAcc      ...  5.010
Radial Grid Type                 RadialGrid  ... Gauss-Chebyshev
Angular Grid (max. acc.)         AngularGrid ... Lebedev-434
Angular grid pruning method      GridPruning ... 3 (G Style)
Weight generation scheme         WeightScheme... Becke
Basis function cutoff            BFCut       ...    1.0000e-11
Integration weight cutoff        WCut        ...    1.0000e-14
Grids for H and He will be reduced by one unit

# of grid points (after initial pruning)     ...  86448 (   0.1 sec)
# of grid points (after weights+screening)   ...  82896 (   0.1 sec)
nearest neighbour list constructed           ...    0.0 sec
```

```
Grid point re-assignment to atoms done       ...    0.0 sec
Grid point division into batches done        ...    1.1 sec
Reduced shell lists constructed in    2.5 sec
Total number of grid points                  ...    82896
Total number of batches                      ...     1299
Average number of points per batch           ...       63
Average number of grid points per atom       ...    10362
Average number of shells per batch           ...    49.15 (55.85%)
Average number of basis functions per batch  ...   141.86 (57.20%)
Average number of large shells per batch     ...    38.02 (77.37%)
Average number of large basis fcns per batch ...   107.64 (75.87%)
Maximum spatial batch extension              ...  17.89, 24.74, 25.35 au
Average spatial batch extension              ...   2.34,  2.47,  2.41 au

Final grid set up in    2.7 sec
Final integration                            ... done (   3.0 sec)
Change in XC energy                          ...     0.000000519
Integrated number of electrons               ...    62.000011559
Previous integrated no of electrons          ...    62.000035465


                      *****************************
                      * MAYER POPULATION ANALYSIS *
                      *****************************

  NA   - Mulliken gross atomic population
  ZA   - Total nuclear charge
  QA   - Mulliken gross atomic charge
  VA   - Mayer's total valence
  BVA  - Mayer's bonded valence
  FA   - Mayer's free valence

  ATOM       NA         ZA         QA         VA         BVA        FA
  0 C      5.6435     6.0000     0.3565     4.7052     4.7052     0.0000
  1 O      8.5526     8.0000    -0.5526     1.8881     1.8881    -0.0000
  2 O      8.5813     8.0000    -0.5813     1.8757     1.8757     0.0000
  3 O      8.2226     8.0000    -0.2226     1.8113     1.8113     0.0000
  4 O      8.2226     8.0000    -0.2226     1.8113     1.8113     0.0000
  5 C      5.6435     6.0000     0.3565     4.7053     4.7053     0.0000
  6 O      8.5526     8.0000    -0.5526     1.8881     1.8881    -0.0000
  7 O      8.5813     8.0000    -0.5813     1.8758     1.8758     0.0000

  Mayer bond orders larger than 0.100000
B(  0-C ,  1-O ) :   1.8511 B(  0-C ,  2-O ) :   1.8181 B(  0-C ,  3-O ) :   0.9710
B(  3-O ,  4-O ) :   0.8031 B(  4-O ,  5-C ) :   0.9710 B(  5-C ,  6-O ) :   1.8511
```

```
B(  5-C ,  7-O ) :   1.8182

Peroxohydrogendicarbonate

               *****************************************************
               *                     SUCCESS                       *
               *           SCF CONVERGED AFTER   6 CYCLES          *
               *****************************************************

Setting up the final grid:

General Integration Accuracy     IntAcc      ...  5.010
Radial Grid Type                 RadialGrid  ... Gauss-Chebyshev
Angular Grid (max. acc.)         AngularGrid ... Lebedev-434
Angular grid pruning method      GridPruning ... 3 (G Style)
Weight generation scheme         WeightScheme... Becke
Basis function cutoff            BFCut       ...    1.0000e-11
Integration weight cutoff        WCut        ...    1.0000e-14
Grids for H and He will be reduced by one unit

# of grid points (after initial pruning)     ...  93140 (   0.1 sec)
# of grid points (after weights+screening)   ...  88738 (   0.2 sec)
nearest neighbour list constructed           ...    0.0 sec
Grid point re-assignment to atoms done       ...    0.0 sec
Grid point division into batches done        ...    1.1 sec
Reduced shell lists constructed in    2.6 sec

Total number of grid points                  ...    88738
Total number of batches                      ...     1392
Average number of points per batch           ...       63
Average number of grid points per atom       ...     9860
Average number of shells per batch           ...    52.59 (55.94%)
Average number of basis functions per batch  ...   148.68 (56.75%)
Average number of large shells per batch     ...    40.34 (76.71%)
Average number of large basis fcns per batch ...   111.83 (75.21%)
Maximum spatial batch extension              ...  18.07, 22.03, 20.89 au
Average spatial batch extension              ...   2.26,  2.32,  2.36 au

Final grid set up in    2.9 sec
Final integration                            ... done (   3.4 sec)
Change in XC energy                          ...    -0.000010793
Integrated number of electrons               ...    62.000000420
Previous integrated no of electrons          ...    62.000005092

----------------
```

```
TOTAL SCF ENERGY
----------------

Total Energy       :         -528.28306509 Eh          -14375.31303 eV

Components:
Nuclear Repulsion  :          380.09058039 Eh           10342.79051 eV
Electronic Energy  :         -908.37364548 Eh          -24718.10354 eV
One Electron Energy:        -1492.48793310 Eh          -40612.66137 eV
Two Electron Energy:          584.11428762 Eh           15894.55783 eV
CPCM Dielectric    :           -0.11068078 Eh              -3.01178 eV

Virial components:
Potential Energy   :        -1054.60183908 Eh          -28697.17498 eV
Kinetic Energy     :          526.31877399 Eh           14321.86195 eV
Virial Ratio       :            2.00373213


DFT components:
N(Alpha)           :       31.000000209926 electrons
N(Beta)            :       31.000000209926 electrons
N(Total)           :       62.000000419851 electrons
E(X)               :      -48.090290841920 Eh
E(C)               :       -2.644421391631 Eh
E(XC)              :      -50.734712233551 Eh
DFET-embed. en.    :        0.000000000000 Eh
CPCM Solvation Model Properties:
Surface-charge          :            0.97742510
Charge-correction       :           -0.00268793 Eh              -0.07314 eV
Free-energy (cav+disp)  :            0.00349333 Eh               0.09506 eV
Corrected G(solv)       :         -528.28225969 Eh          -14375.29111 eV


                      *****************************
                      * MAYER POPULATION ANALYSIS *
                      *****************************

  NA   - Mulliken gross atomic population
  ZA   - Total nuclear charge
  QA   - Mulliken gross atomic charge
  VA   - Mayer's total valence
  BVA  - Mayer's bonded valence
  FA   - Mayer's free valence

  ATOM       NA         ZA         QA         VA         BVA        FA
```

```
  0 C      5.5571     6.0000     0.4429     4.5121     4.5121    -0.0000
  1 O      8.2632     8.0000    -0.2632     2.1944     2.1944    -0.0000
  2 O      8.4070     8.0000    -0.4070     2.0958     2.0958     0.0000
  3 O      8.1512     8.0000    -0.1512     2.0593     2.0593     0.0000
  4 O      8.2131     8.0000    -0.2131     1.7647     1.7647    -0.0000
  5 C      5.6387     6.0000     0.3613     4.6501     4.6501    -0.0000
  6 O      8.5110     8.0000    -0.5110     1.9472     1.9472     0.0000
  7 O      8.5286     8.0000    -0.5286     1.9519     1.9519     0.0000
  8 H      0.7302     1.0000     0.2698     0.9836     0.9836     0.0000

  Mayer bond orders larger than 0.100000
B(  0-C ,  1-O ) :   1.2382 B(  0-C ,  2-O ) :   1.9919 B(  0-C ,  3-O ) :   1.1737
B(  1-O ,  8-H ) :   0.9268 B(  3-O ,  4-O ) :   0.7975 B(  4-O ,  5-C ) :   0.8860
B(  5-C ,  6-O ) :   1.8753 B(  5-C ,  7-O ) :   1.8505

Peroxide

               *****************************************************
               *                     SUCCESS                       *
               *           SCF CONVERGED AFTER   3 CYCLES          *
               *****************************************************

Setting up the final grid:

General Integration Accuracy     IntAcc      ...  5.010
Radial Grid Type                 RadialGrid  ... Gauss-Chebyshev
Angular Grid (max. acc.)         AngularGrid ... Lebedev-434
Angular grid pruning method      GridPruning ... 3 (G Style)
Weight generation scheme         WeightScheme... Becke
Basis function cutoff            BFCut       ...    1.0000e-11
Integration weight cutoff        WCut        ...    1.0000e-14
Grids for H and He will be reduced by one unit

# of grid points (after initial pruning)     ...  21684 (   0.0 sec)
# of grid points (after weights+screening)   ...  21656 (   0.0 sec)
nearest neighbour list constructed           ...    0.0 sec
Grid point re-assignment to atoms done       ...    0.0 sec
Grid point division into batches done        ...    0.9 sec
Reduced shell lists constructed in    1.0 sec

Total number of grid points                  ...    21656
Total number of batches                      ...      340
Average number of points per batch           ...       63
Average number of grid points per atom       ...    10828
Average number of shells per batch           ...    15.63 (71.05%)
```

```
Average number of basis functions per batch  ...     46.61 (75.18%)
Average number of large shells per batch     ...     14.14 (90.47%)
Average number of large basis fcns per batch ...     41.92 (89.93%)
Maximum spatial batch extension              ...  17.04, 15.15, 16.57 au
Average spatial batch extension              ...   3.06,  3.09,  3.06 au

Final grid set up in    1.1 sec
Final integration                            ... done (   0.4 sec)
Change in XC energy                          ...    -0.000010961
Integrated number of electrons               ...    17.999999563
Previous integrated no of electrons          ...    17.999993496

----------------
TOTAL SCF ENERGY
----------------

Total Energy       :         -150.52414932 Eh           -4095.97034 eV

Components:
Nuclear Repulsion  :           21.97216652 Eh             597.89305 eV
Electronic Energy  :         -172.49631584 Eh           -4693.86339 eV
One Electron Energy:         -262.39468878 Eh           -7140.12248 eV
Two Electron Energy:           89.89837293 Eh            2446.25909 eV
CPCM Dielectric    :           -0.49899231 Eh             -13.57827 eV

Virial components:
Potential Energy   :         -300.48982653 Eh           -8176.74388 eV
Kinetic Energy     :          149.96567720 Eh            4080.77354 eV
Virial Ratio       :            2.00372400


DFT components:
N(Alpha)           :        8.999999781471 electrons
N(Beta)            :        8.999999781471 electrons
N(Total)           :       17.999999562942 electrons
E(X)               :      -13.412494335279 Eh
E(C)               :       -0.742340404234 Eh
E(XC)              :      -14.154834739514 Eh
DFET-embed. en.    :        0.000000000000 Eh
CPCM Solvation Model Properties:
Surface-charge          :            1.96870204
Charge-correction       :           -0.00793286 Eh              -0.21586 eV
Free-energy (cav+disp)  :            0.00268317 Eh               0.07301 eV
Corrected G(solv)       :         -150.52939902 Eh           -4096.11319 eV
```

```
                    ****************************
                    * MAYER POPULATION ANALYSIS *
                    ****************************

  NA   - Mulliken gross atomic population
  ZA   - Total nuclear charge
  QA   - Mulliken gross atomic charge
  VA   - Mayer's total valence
  BVA  - Mayer's bonded valence
  FA   - Mayer's free valence

  ATOM       NA         ZA         QA         VA         BVA        FA
  0 O      9.0000     8.0000    -1.0000     0.8295     0.8295    -0.0000
  1 O      9.0000     8.0000    -1.0000     0.8295     0.8295     0.0000

  Mayer bond orders larger than 0.100000
B(  0-O ,  1-O ) :   0.8295
```

5 Analysis

5.1 Peroxodicarbonate

Oxidant concentration was determined by iodometric titration where the oxidants H_2O_2 and $Na_2C_2O_6$ oxidize iodide to iodine in the presence of sulphuric acid and with molybdate as catalyst.

$$Na_2C_2O_6 + 2\,H_2SO_4 + 2\,KI \longrightarrow I_2 + K_2SO_4 + Na_2SO_4 + 2\,H_2O + 2\,CO_2 \tag{1}$$

$$H_2O_2 + H_2SO_4 + 2\,KI \longrightarrow I_2 + K_2SO_4 + 2\,H_2O \tag{2}$$

$$I_2 + 2\,Na_2S_2O_3 \longrightarrow Na_2S_4O_6 + 2\,NaI \tag{3}$$

Initially, a 5 ml sample solution was mixed with 10 ml of a 20% (v/v) H_2SO_4 solution. Subsequently, an excess of KI solution was added, resulting in the oxidation of iodide to brownish iodine. To identify the end point, a few drops of a 1% (w/v) starch solution were employed. Additionally, a catalyst in the form of a few drops of a 3% ammonium molybdate tetra-hydrate solution was introduced. Finally, the total oxidant content in the solution was titrated with sodium thiosulfate until discolouration occurs.

5.2 Components and their yields derived from lignin

5.2.1 Work-up procedure

Depending on the concentration of the reactor product, for the extraction, a 5-20 ml sample of reactor product was acidified to pH 1 using concentrated HCl under constant stirring. The solution was allowed to equilibrate for one hour. The extraction was executed by adding 10-20 ml ethyl acetate under continuous stirring, extraction time 10 min. The organic layer was decanted and collected in a Schott vessel. This procedure was repeated 4 times and the total extract volume was collected and combined. These combined organic extracts were then dried with anhydrous $MgSO_4$. An aliquot of 10 ml was evaporated at 30 ^oC with a SBHCONC/1 sample concentrator (Stuart) and at a gentle stream of pressurized air (approx. 5 psi) to gravimetrically determine the dry mass of the extract (mass recovery).

5.2.2 GS-MS screening and quantification

GC-MS (single quadrupole) was used for analysis (Agilent 7890A). The injection volume was 0.5 µl. Split injection was chosen at a ratio of 5:1. Carrier gas was He at a flow rate of 1.8 ml/min. Analysis was carried out on an HP-5MS column (Agilent Technologies, 30 m x 250 µm x 0.25 µm). The temperature program was set as follows: Starting at 40 °C, hold for 5.66 min, then 8.8 °C/min to 100 °C for 1.7 min, then 13.3 °C/min to 220 °C for 3.39 min, then 10 °C/min to 325 °C for 3.43 min. The total analysis time was 40.521 min. MSD transfer line temperature was 230 °C. The solvent delay was set to 4.00 min.

The compound screening was done by using the software package "Unknown Analysis/Quant-my-way" Agilent Masshunter. The spectra of the analyzed samples were compared with the spectra of known compounds stored in the National Institute Standard and Technology database, which contains more than 62,000 samples.

List of phenolic monomers identified by using the NIST library:

- 2-Cyclopenten-1-one, 3,4-dimethyl-
- 2-Cyclopenten-1-one, 2-methyl-
- 1-Pentanone, 1-(2-furanyl)-
- Phenol, 2-methoxy-
- Benzaldehyde
- Acetophenone
- 2-Thiophenecarboxaldehyde
- 5-Methyl-2-thiophenecarboxaldehyde
- 1-Pentanone, 1-(2-thienyl)
- 1,2-Ethanediol, 1,2-diphenyl-
- p-Cresol
- Phenol
- Phenol, 4-ethyl-2-methoxy-
- Ethanone, 1-(2-hydroxy-5-methylphenyl)-
- Vanillin
- Apocynin (Acetovanillone)
- 2-Propanone, 1-(4-hydroxy-3-methoxyphenyl)-

- 4-Ethoxy-3-anisaldehyde
- Benzeneacetic acid, 3,4-dimethoxy-
- Homovanillyl alcohol
- Homovanillic acid
- 2-Butanone, 4-(4-hydroxy-3-methoxyphenyl)-

Qualitative analysis was performed using d3-vanillin as an internal standard. In addition, the standards of the target analytes were assayed. A stock solution of the 11 target analytes was prepared and dissolved at 1 mg/mL in ethanol and stored in amber glass vials at 4 °C in the fridge until further analyses. The limit of quantification (LLOQ) was set as the lowest acceptable value from the calibration solution. The limit of detection (LOD) was estimated as LLOQ/3.[29] Target analyte quantification was carried out on the basis of the internal standard method using d3-Vanillin as the internal standard at a concentration of 50 ug mL^{-1}.

The accuracy and precision of the method were assessed through recovery and repeatability experiments, respectively. Three target analytes (Guaiacol, Vanillin, and Acetovanillone) were selected. Four replicate analyses (N=4) were prepared. Relative recoveries calculated according to Asimakopoulos et al.[29] In summary, relative recoveries of target analytes were ranging between 73 - 98%. Intra-Day precision and inter-Day precision were <10% RSD. The linearity of the method was examined by solvent calibration standards at 5 - 6 concentration levels, ranging between 0.5 - 100 ug mL^{-1}, depending on estimated LLOQs. The regression coefficients of all calibration curves were $\geq$0.99. Possible contamination resulting during sample treatment was assessed by procedural blanks. Solvent blanks (ethyl acetate) were frequently injected to record potential carry-over and cross-contamination in the instrument.

6 Modelling

Table S7 shows experimental data from the pilot which is used for tuning the steady state model parameters. Base case parameters for this system are displayed in Table S8.

Table S7: Experimental data from the Tiller pilot, steady-state data. Constant operating conditions: temperature after cooler T = 11 ^oC; sodium carbonate feed concentration $c_{F,Na_2CO_3} = 1$ M; Circulation flow rate $v_E = 14.5$ l/min. Steady-state is defined as a sample taken longer than 3 hours since the last change in set-point.

Id	date<br>yy-mm-dd	v_P<br>l/h	ρ_I<br>mA/cm^2	$c_{R,Na_2C_2O_6}$<br>M	U<br>V	$\epsilon_{peroxodicarbonate}$<br>%	ϵ_U<br>%
POD0004	2021-07-16	10.7	400	0.083	5.59	3.2	0.58
POD0005	2021-07-16	10.7	400	0.103	5.59	-16.8	0.58
POD0006	2021-07-16	10.7	400	0.100	5.59	-14.3	0.58
POD0007	2021-07-16	10.7	400	0.097	5.59	-11.7	0.58
POD0009	2021-08-17	4.4	675	0.188	6.51	-3.7	2.21
POD0010	2021-08-17	4.4	675	0.165	6.51	9.7	2.21
POD0013	2021-08-20	4.4	400	0.130	5.59	0.4	0.58
POD0015	2021-09-01	4.4	400	0.125	5.59	4.4	0.58
POD0016	2021-09-01	4.4	400	0.120	5.59	8.8	0.58
POD0021	2022-02-10	10.3	675	0.160	6.71	9.0	-0.83
POD0022	2022-02-10	10.7	675	0.170	6.73	-0.03	-1.13
POD0023	2022-02-10	10.3	675	0.170	6.75	2.6	-1.42
POD0024	2022-02-10	10.3	675	0.183	6.74	-4.7	-1.27
POD0025	2022-02-10	10.9	675	0.166	6.73	1.4	-1.13
POD0031	2022-02-11	4.1	675	0.200	6.71	-12.7	-0.83
POD0038	2022-05-05	10.7	200	0.034	4.92	10.7	-0.98
POD0039	2022-05-05	10.7	200	0.035	4.93	6.9	-1.18
POD0044	2022-05-10	4.7	200	0.060	4.87	-7.5	0.04
POD0045	2022-05-10	4.3	200	0.066	4.87	-13.3	0.04
POD0046	2022-05-10	1.9	200	0.067	4.87	8.0	0.04

The fit of the model is fairly scattered with respect to the peroxodicarbonate concentration, whereas the prediction of terminal cell voltage is more accurate (Figure S6). This likely indicates uncertainty in the experimental data.

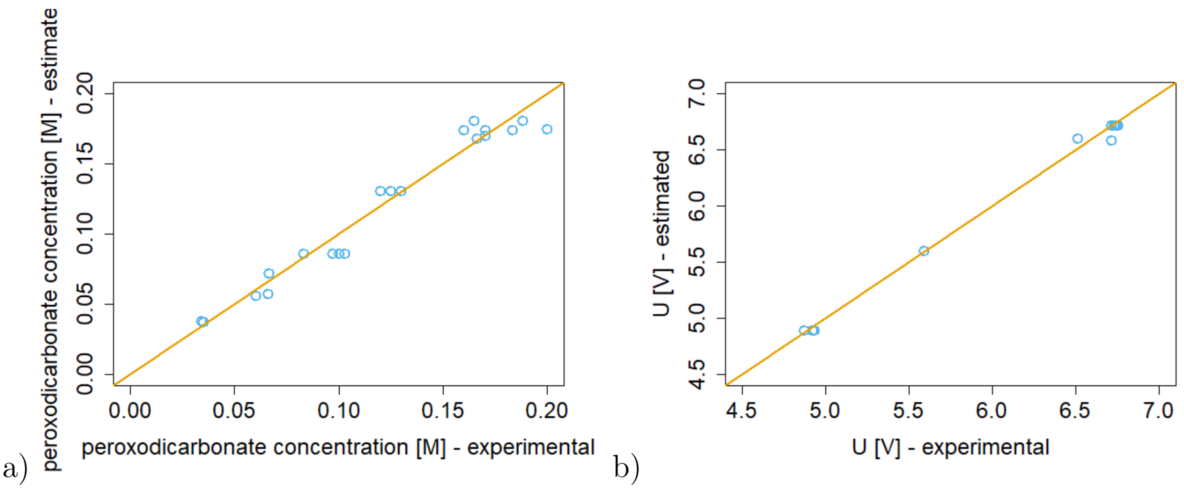

a) b)

Figure S6: Comparison (actual deviations) of model prediction and experimental data for a) peroxodicarbonate concentration, $c_{R,PODIC}$ and b) terminal cell voltage, U.

Table S8: Base case operating conditions, system dimensions, and model parameters.

Parameter	Description	Value	Unit
	**Base case operation conditions:**		
$c_{F,\mathrm{Na_2CO_3}}$	Feed concentration	1	M
ρ_I	Current density	675	mA/cm2
T	Temperature	284	K
P	Pressure	101	kPa
v_E	Circulation rate	12.5	l/min
v_P	Product rate	10	l/h
	**System dimensions:**		
a	Electrode area	435	cm2
l	Electrode distance	0.0015	m
w	Electrode width	0.3	m
V	Liquid holdup	10	l
	**Model parameters:**		
A_2	Frequency factor peroxodicarbonate decomposition	70	1/s
E_2	Activation energy peroxodicarbonate decomposition	3e+04	J/(mol*K)
keff	keff in eff=keff + meff*rhoI	0.36	-
meff	meff in eff=keff + meff*rhoI	0.00031	cm2/mA
kU	kU in U = kU + rhoI*l/(k0E*(1-eG)**1.5)	4.2	V
k0E	k0E in U = kU + rhoI*l/(k0E*(1-eG)**1.5)	0.48	-

Solving the least square minimization gives the following model parameters, which all are found to significantly describe the variance in the model data:

```
      Estimates  Std.error    t-value      Pr(>|t|) Sign
keff   0.363209   0.023410  15.515128  0.000000e+00  ***
meff   0.000312   0.000044   7.033324  3.460487e-08  ***
A2    70.347258   4.000065  17.586530  0.000000e+00  ***
kU     4.237817   0.193250  21.929244  0.000000e+00  ***
k0E    0.476760   0.057709   8.261518  9.744932e-10  ***
---
Signif. codes:  0 ‘***’ 0.001 ‘**’ 0.01 ‘*’ 0.05 ‘.’ 0.1 ‘ ’ 1
Residual standard error: 0.06722 on 35 degrees of freedom
```

Parameter correlation matrix:

```
      keff  meff   A2    kU    k0E
keff  1.00 -0.72  0.60 -0.01 -0.001
meff -0.72  1.00  0.002 0.02  0.03
A2    0.60  0.002 1.00  0.01  0.03
kU   -0.01  0.02  0.01  1.00  0.90
k0E  -0.001 0.03  0.03  0.90  1.00
```

References

(1) Neese, F. The ORCA program system. *WIREs Computational Molecular Science* **2012**, *2*, 73–78, doi: 10.1002/wcms.81.

(2) Weigend, F.; Ahlrichs, R. Balanced basis sets of split valence, triple zeta valence and quadruple zeta valence quality for H to Rn: Design and assessment of accuracy. *Physical Chemistry Chemical Physics* **2005**, *7*, 3297–3305, doi: 10.1039/B508541A.

(3) Schäfer, A.; Huber, C.; Ahlrichs, R. Fully optimized contracted Gaussian basis sets of triple zeta valence quality for atoms Li to Kr. *The Journal of Chemical Physics* **1994**, *100*, 5829–5835.

(4) Eichkorn, K.; Weigend, F.; Treutler, O.; Ahlrichs, R. Auxiliary basis sets for main row atoms and transition metals and their use to approximate Coulomb potentials. *Theoretical Chemistry Accounts* **1997**, *97*, 119–124, doi: 10.1007/s002140050244.

(5) Weigend, F.; Furche, F.; Ahlrichs, R. Gaussian basis sets of quadruple zeta valence quality for atoms H–Kr. *The Journal of Chemical Physics* **2003**, *119*, 12753–12762, doi: 10.1063/1.1627293.

(6) Schäfer, A.; Horn, H.; Ahlrichs, R. Fully optimized contracted Gaussian basis sets for atoms Li to Kr. *The Journal of Chemical Physics* **1992**, *97*, 2571–2577, doi: 10.1063/1.463096.

(7) Metz, B.; Stoll, H.; Dolg, M. Small-core multiconfiguration-Dirac–Hartree–Fock-adjusted pseudopotentials for post-d main group elements: Application to PbH and PbO. *The Journal of Chemical Physics* **2000**, *113*, 2563–2569, doi: 10.1063/1.1305880.

(8) Dirac, P. A. M.; Fowler, R. H. Quantum mechanics of many-electron systems. *Proceedings of the Royal Society of London. Series A, Containing Papers of a Mathematical and Physical Character* **1997**, *123*, 714–733, doi: 10.1098/rspa.1929.0094.

(9) Slater, J. C. A Simplification of the Hartree-Fock Method. *Physical Review* **1951**, *81*, 385–390, doi: 10.1103/PhysRev.81.385.

(10) Vosko, S. H.; Wilk, L.; Nusair, M. Accurate spin-dependent electron liquid correlation energies for local spin density calculations: a critical analysis. *Canadian Journal of Physics* **1980**, *58*, 1200–1211, doi: 10.1139/p80-159.

(11) Becke, A. D. Density-functional exchange-energy approximation with correct asymptotic behavior. *Physical Review A* **1988**, *38*, 3098–3100, doi: 10.1103/PhysRevA.38.3098.

(12) Lee, C.; Yang, W.; Parr, R. G. Development of the Colle-Salvetti correlation-energy formula into a functional of the electron density. *Physical Review B* **1988**, *37*, 785–789, doi: 10.1103/PhysRevB.37.785.

(13) Becke, A. D. Density-functional thermochemistry. III. The role of exact exchange. *Journal of Chemical Physics* **1993**, *98*, 5648–5652, doi: 10.1063/1.464913.

(14) Graßl, T. Synthese und Charakterisierung von C60-Fulleriden, Untersuchung der Reaktion von Kohlenhydraten in flüssigem Ammoniak und Umsetzung von Heptapnictiden (E = P, As) mit Ethinen und Gruppe-12-Metallkomplexen. Ph.D. thesis, Universität Regensburg, Regensburg, 2016.

(15) Savin, A.; Nesper, R.; Wengert, S.; Fässler, T. F. Die Elektronenlokalisierungsfunktion ELF. *Angewandte Chemie* **1997**, *109*, 1892–1918, doi: 10.1002/ange.19971091706.

(16) Savin, A. On the significance of ELF basins. *Journal of Chemical Sciences* **2005**, *117*, 473–475, doi: 10.1007/BF02708351.

(17) Becke, A. D.; Edgecombe, K. E. A simple measure of electron localization in atomic and molecular systems. *Journal of Chemical Physics* **1990**, *92*, 5397–5403, doi: 10.1063/1.458517.

(18) Savin, A.; Becke, A. D.; Flad, J.; Nesper, R.; Preuss, H.; von Schnering, H. G. A New Look at Electron Localization. *Angewandte Chemie International Edition in English* **1991**, *30*, 409–412, _eprint: https://onlinelibrary.wiley.com/doi/pdf/10.1002/anie.199104091.

(19) Silvi, B.; Savin, A. Classification of chemical bonds based on topological analysis of electron localization functions. *Nature* **1994**, *371*, 683–686, doi: 10.1038/371683a0.

(20) Fässler, T. F.; Savin, A. Chemische Bindung anschaulich: die Elektronen-Lokalisierungs-Funktion. *Chemie in unserer Zeit* **1997**, *31*, 110–120, doi: 10.1002/ciuz.19970310303.

(21) Burdett, J. K.; McCormick, T. A. Electron Localization in Molecules and Solids: The

Meaning of ELF. *The Journal of Physical Chemistry A* **1998**, *102*, 6366–6372, doi: 10.1021/jp9820774.

(22) Tsirelson, V.; Stash, A. Determination of the electron localization function from electron density. *Chemical Physics Letters* **2002**, *351*, 142–148, doi: 10.1016/S0009-2614(01)01361-6.

(23) Chamorro, E.; Fuentealba, P.; Savin, A. Electron probability distribution in AIM and ELF basins. *Journal of Computational Chemistry* **2003**, *24*, 496–504, doi: 10.1002/jcc.10242.

(24) Silvi, B.; Fourré, I.; Alikhani, M. E. The Topological Analysis of the Electron Localization Function. A Key for a Position Space Representation of Chemical Bonds. *Monatshefte für Chemie / Chemical Monthly* **2005**, *136*, 855–879, doi: 10.1007/s00706-005-0297-8.

(25) Silvi, B. How topological partitions of the electron distributions reveal delocalization. *Physical Chemistry Chemical Physics* **2004**, *6*, 256–260, doi: 10.1039/B311272A.

(26) Bader, R. The Quantum Mechanical Basis of Conceptual Chemistry. *Monatshefte für Chemie - Chemical Monthly* **2005**, *136*, 819–854, doi: 10.1007/s00706-005-0307-x.

(27) Poater, J.; Duran, M.; Solà, M.; Silvi, B. Theoretical Evaluation of Electron Delocalization in Aromatic Molecules by Means of Atoms in Molecules (AIM) and Electron Localization Function (ELF) Topological Approaches. *Chemical Reviews* **2005**, *105*, 3911–3947, doi: 10.1021/cr030085x.

(28) Lu, T.; Chen, F. Multiwfn: A multifunctional wavefunction analyzer. *Journal of Computational Chemistry* **2012**, *33*, 580–592, doi: 10.1002/jcc.22885.

(29) Asheim, J.; Vike-Jonas, K.; Gonzalez, S. V.; Lierhagen, S.; Venkatraman, V.; Veivåg, I.-L. S.; Snilsberg, B.; Flaten, T. P.; Asimakopoulos, A. G. Benzotriazoles, benzothiazoles

and trace elements in an urban road setting in Trondheim, Norway: Re-visiting the chemical markers of traffic pollution. *Science of The Total Environment* **2019**, *649*, 703–711, doi: 10.1016/j.scitotenv.2018.08.299.
